# Engineering the acyltransferase domain of epothilone polyketide synthase to alter the substrate specificity

**DOI:** 10.1186/s12934-021-01578-3

**Published:** 2021-04-21

**Authors:** Huimin Wang, Junheng Liang, Qianwen Yue, Long Li, Yan Shi, Guosong Chen, Yue-zhong Li, Xiaoying Bian, Youming Zhang, Guoping Zhao, Xiaoming Ding

**Affiliations:** 1 Collaborative Innovation Center for Genetics and Development, State Key Laboratory of Genetic Engineering, Shanghai Engineering Research Center of Industrial Microorganisms, Department of Microbiology, School of Life Sciences, Fudan University, Shanghai, 200438 People’s Republic of China; 2 The State Key Laboratory of Molecular Engineering of Polymers, Department of Macromolecular Science, Fudan University, Shanghai, 200433 People’s Republic of China; 3 State Key Laboratory of Microbial Technology, School of Life Sciences, Shandong University-Helmholtz Institute of Biotechnology, Shandong University, Qingdao, Shandong People’s Republic of China; 4 CAS Key Laboratory of Synthetic Biology, Institute of Plant Physiology and Ecology, Shanghai Institutes for Biological Sciences, Chinese Academy of Sciences, Shanghai, 200032 People’s Republic of China

**Keywords:** Polyketide synthase, AT, Epothilone, Domain swap, Substrate specificity

## Abstract

**Background:**

Polyketide synthases (PKSs) include ketone synthase (KS), acyltransferase (AT) and acyl carrier protein (ACP) domains to catalyse the elongation of polyketide chains. Some PKSs also contain ketoreductase (KR), dehydratase (DH) and enoylreductase (ER) domains as modification domains. Insertion, deletion or substitution of the catalytic domains may lead to the production of novel polyketide derivatives or to the accumulation of desired products. Epothilones are 16-membered macrolides that have been used as anticancer drugs. The substrate promiscuity of the module 4 AT domain of the epothilone PKS (EPOAT4) results in production of epothilone mixtures; substitution of this domain may change the ratios of epothilones. In addition, there are two dormant domains in module 9 of the epothilone PKS. Removing these redundant domains to generate a simpler and more efficient assembly line is a desirable goal.

**Results:**

The substitution of module 4 drastically diminished the activity of epothilone PKS. However, with careful design of the KS-AT linker and the post-AT linker, replacing EPOAT4 with EPOAT2, EPOAT6, EPOAT7 or EPOAT8 (specifically incorporating methylmalonyl-CoA (MMCoA)) significantly increased the ratio of epothilone D (**4**) to epothilone C (**3**) (the highest ratio of **4**:**3** = 4.6:1), whereas the ratio of **4**:**3** in the parental strain *Schlegelella brevitalea* 104-1 was 1.4:1. We also obtained three strains by swapping EPOAT4 with EPOAT3, EPOAT5, or EPOAT9, which specifically incorporate malonyl-CoA (MCoA). These strains produced only epothilone C, and the yield was increased by a factor of 1.8 compared to that of parental strain 104-1. Furthermore, mutations of five residues in the AT domain identified Ser310 as the critical factor for MMCoA recognition in EPOAT4. Then, the mutation of His308 to valine or tyrosine combined with the mutation of Phe310 to serine further altered the product ratios. At the same time, we successfully deleted the inactive module 9 DH and ER domains and fused the ΨKR domain with the KR domain through an ~ 25-residue linker to generate a productive and simplified epothilone PKS.

**Conclusions:**

These results suggested that the substitution and deletion of catalytic domains effectively produces desirable compounds and that selection of the linkers between domains is crucial for maintaining intact PKS catalytic activity.

**Supplementary Information:**

The online version contains supplementary material available at 10.1186/s12934-021-01578-3.

## Background

Nonribosomal peptides (NRPs) and polyketides (PKs) are two prominent families of natural products, some of which exhibit high bioactivity [1, 2]. Nonribosomal peptide synthetases (NRPSs) and polyketide synthases (PKSs) assemble amino acids and simple acyl-CoA building blocks into the final complex compounds NRPs and PKs, respectively [3–5]. Each PKS module contains three necessary domains, the ketosynthase (KS) domain, acyltransferase (AT) domain, and acyl carrier protein (ACP) domain [6–8]. Some modules also contain optional processing domains, a ketoreductase (ΨKR/KR) domain, a dehydratase (DH) domain, and an enoylreductase (ER) domain that are used for successively catalysing the reduction of the resulting β-keto group [9, 10]. Notably, among processing domains, ΨKR acts not as a catalytic domain but as a structural domain to support the integrity of the active site of the catalytic KR domain [11]. The thioesterase (TE) domain releases the eventual polyketide compounds. The collinearity (strict correlation of the PKS organization and the product structure) of PKSs has sparked interest in the generation of new derivatives by PKS engineering [12–16].

Epothilones are members of the NRP/PK family synthesized by hybrid NRPS/PKS synthetases, which stabilize microtubules in parallel with taxol and have been used as anticancer agents [17]. The natural producer myxobacterium *Sorangium cellulosum* produces epothilone A and B, while epothilone C and D are produced by inactivation or deletion of *epoK*, which is an epoxidase gene in the epothilone biosynthesis gene cluster (MIBiG-ID BGC0000989) [18]. Epothilone B and D were proved to have higher activity than epothilone A and C [19]. The marketed anticancer drug ixabepilone (IXEMPRA™) is a Food and Drug Administration (FDA)-approved synthetic analogue of epothilone B, and utidelone (epothilone D) could effectively treat breast cancer in phase 3 trials [20, 21]. Mixtures of epothilones were generated due to the module 4 native AT domain of the epothilone PKS (EPOAT4), which can add either methylmalonyl-CoA (MMCoA) or malonyl-CoA (MCoA) extender units at positions C11–C12 during polyketide chain elongation [22]. We wanted to engineer EPOAT4 for two main reasons. First, the production of mixtures of compounds is a disadvantage in industrial production, and changing the ratios to favour preferred compounds has commercial potential. Second, the unique substrate specificity of EPOAT4 makes it an excellent model for the comparison of MMCoA and MCoA incorporation *in vivo* to improve our understanding of the AT domain. The AT engineering mainly include three strategies: swapping domains, exchanging modules, and site-directed mutagenesis of PKS genes. EPOAT4 has been used as a donor AT in the engineering of 6-dEB synthase (DEBS) [22, 23], but to date, EPOAT4 has not been engineered in epothilone PKS *in vivo*. The genetic manipulation in the original epothilone producer is difficult. In previous studies, epothilone PKS was modified in heterologous host *Myxococcus xanthus* by the inactivation of KR, DH or ER domains in EpoD, resulted in production of novel epothilone derivatives but in lower yields for use in industrial production [18, 24, 25]. Moreover, engineering AT domains resulted in novel polyketides but usually lower yields in early studies due to a lack of understanding of the structure of AT [26–29].

**Fig. 1:**
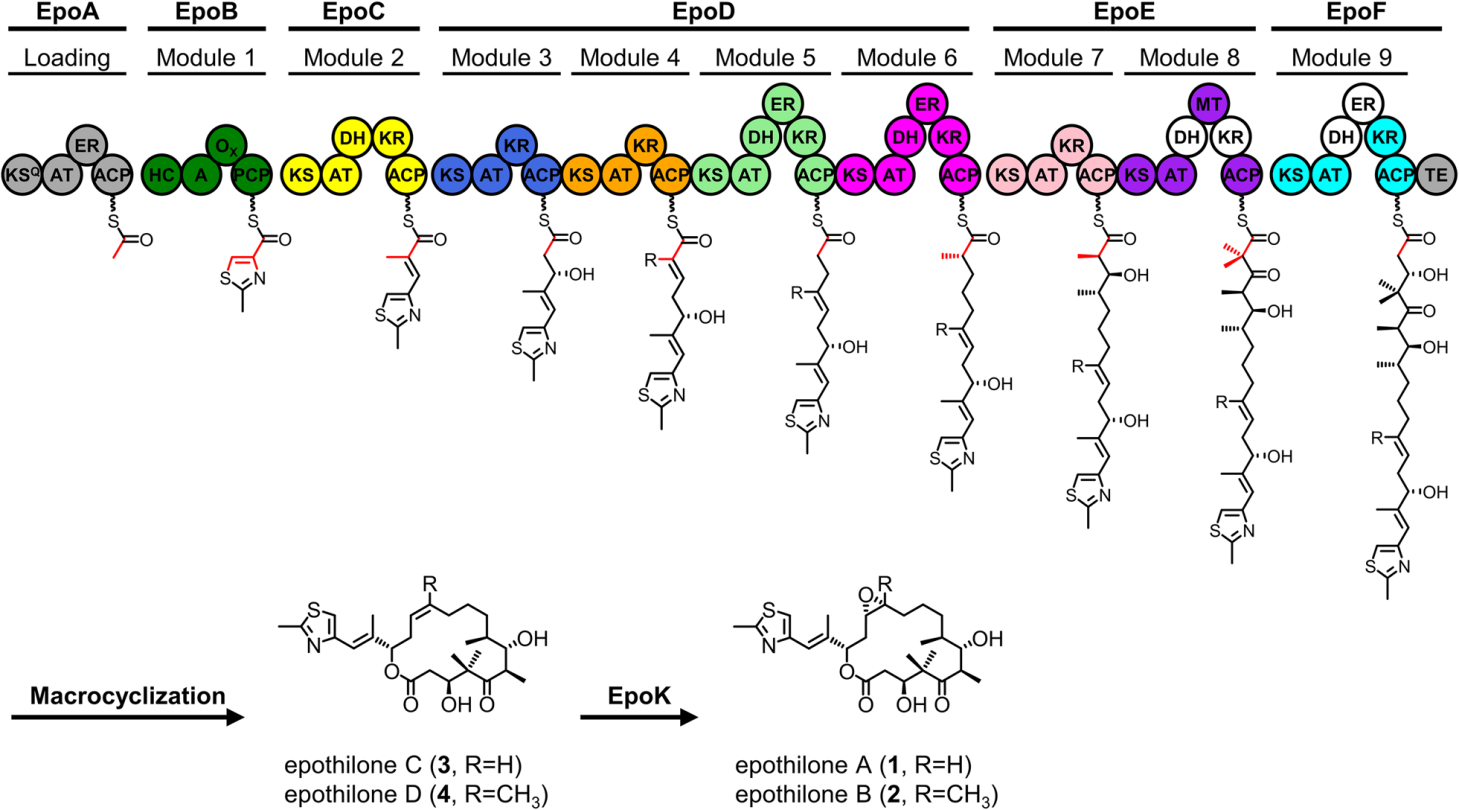
Module organization of epothilone synthase and structures of epothilones A–D. The modular architecture of the six constituent proteins (EpoA–F) is shown in cartoon form. Domain annotation: *KS* ketosynthase; *AT* acyltransferase; *ACP* acyl carrier protein; *MT* methyltransferase; *KR* ketoreductase; *DH* dehydratase; *ER* enoylreductase; *TE* thioesterase. The biosynthetic building blocks derived from L-cysteine, *S*-adenosylmethionine (*S*AM), acetyl-CoA, (2*S*)-methylmalonyl-CoA, and malonyl-CoA are highlighted with red lines. Empty circles indicate nonfunctional domains. Epothilones C and D are converted to epothilones A and B, respectively, by EpoK

Moreover, module 9 of the epothilone PKS (EPOM9) is a sufficiently reducing module with a complete modifying region, and we observed that the DH and ER domains of module 9 (EPODH9 and EPOER9) are inactive (Fig. 1). Inactive domains are common in PKSs but often ignored; for example, there are five dormant domains in rapamycin PKS [30] and six inactive domains in rifamycin PKS [31]. We speculated that the deletion of these redundant domains could reduce the size of the epothilone gene cluster and might simultaneously improve the efficiency of epothilone PKS. We also used EPOM9 as a model to explore whether the inactive processing domains could be removed freely, providing an engineering strategy for other PKSs. Unlike the partly reducing module (KS-AT-ΨKR-KR-ACP), the catalytic KR domain and non-catalytic ΨKR are split by the ER domain in fully reducing modules (KS-AT-DH-ΨKR-ER-KR-ACP). Structural information shows that the ΨKR/KR domains are laterally tethered, that there is an absence of stable interfaces between the different reductive domains, and that the KR domain does not contact its adjacent domains [32] (Additional file 1: Figure S1a). These facts suggest the possibility of deleting inactive EPODH9 and EPOER9 without affecting the activity of epothilone PKS.

In our previous work, we successfully expressed epothilone PKS in the heterologous host *Schlegelella brevitalea* DSM 7029 and obtained mutant strains that produced high levels of epothilone C and D by the deletion of *epoK* [33]. The high epothilone yields and facile genetic manipulation in our heterologous host provide us a platform to engineer epothilone PKS. Herein, we rationally engineered domains in module 4 of the epothilone PKS (EPOM4) and EPOM9 and constructed a series of hybrid epothilone PKSs in the heterologous epothilone producer *S. brevitalea*. We significantly increased the proportion of epothilone D and obtained three strains that produced only epothilone C. We also successfully deleted the inactive DH and ER domains in EPOM9 and fused ΨKR with KR through an ~ 25-residue linker to generate a productive epothilone PKS and simplified the completely reducing region to a partly reducing region in EPOM9 without a decrease in the production level. Finally, our study further explored the importance and the sequence flexibility of ΨKR-KR-linker.

## Results

### The turnover rates of chimaeric epothilone PKSs designed by module 4 exchanges were drastically diminished

Engineering the AT specificity can alter the carbon skeleton of the polyketide by selecting different acyl-CoA substrates. To compare three strategies in altering AT specificity, we first exchanged the module in which EPOAT4 is located. The previously optimized high-yield epothilone producer *S. brevitalea* 104-1 produced 27.7 mg/l epothilone D (**4**) and 19.9 mg/l epothilone C (**3**) (**4**:**3** = 1.4:1) as a consequence of EPOAT4 incorporating either MMCoA or MCoA extender units during polyketide chain elongation [34]. We exchanged EPOM4 with modules that harbour MMCoA-specific AT domains. Four chimaeric epothilone PKSs were constructed in which EPOM4 was replaced by module 7 of the epothilone PKS (EPOM7), module 6 from the erythromycin PKS (ERYM6), and module 4 or 10 from the rapamycin PKS (RAPM4 and RAPM10) (Additional file 1: Figure S2). To preserve interactions between modules related to the catalytic integrity of the epothilone PKS, EPOM4 was replaced by EPOM7 in a manner that retained the natural intermodular linker between EPOM3 and EPOM4 and between EPOM7 and EPOM8 to generate MMR2044 (EPOM7) (Additional file 1: Table S1). No downstream modules were connected to the modules for the other three fusions, MMR2024 (ERYM6), MMR2027 (RAPM4), and MMR2026 (RAPM10), through covalent linkers in their natural polypeptides. Thus, in these three mutants, EPOM3, the heterologous modules and EPOM5 were joined by two covalent linkers from EpoD (Additional file 1: Table S1). However, the production of epothilones in MMR2027 and MMR2026 was completely abolished. MMR2024 and MMR2044 yielded detectable amounts of epothilone C and D, as shown by LC–MS (Additional file 1: Figure S3), but the yields were quite low. MMR2024 produced only 4 µg/l epothilone C and 14 µg/l epothilone D. MMR2044 produced 15 µg/l epothilone C and 17 µg/l epothilone D (Additional file 1: Table S2).

### **EPOAT4 domain swapping alters the ratio of epothilone D to epothilone C**

**Fig. 2:**
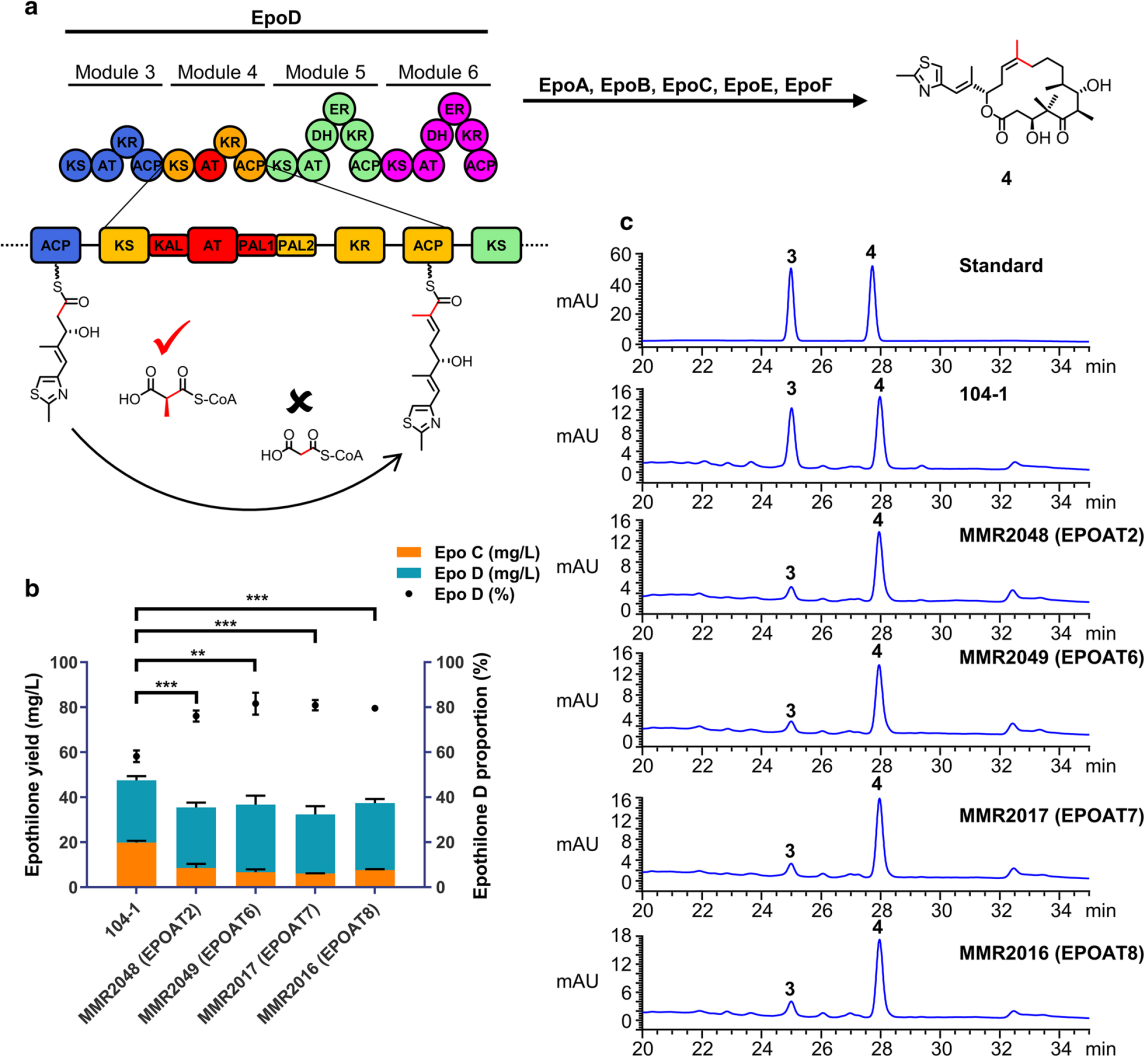
Replacing EPOAT4 with the AT domains specific to MMCoA can significantly increase the ratio of epothilone D to epothilone C. **a** The module 4 AT domain of epothilone PKS (EPOAT4) was replaced with MMCoA-specific AT domains (shown in red). Domain annotation: KAL, KS to AT linker; PAL1, non-conserved N-terminal region in the post-AT linker; PAL2, conserved C-terminal region in the post-AT linker. **b** Epothilone production and epothilone D proportion from six engineered EPOAT4-swapped mutant strains (MMR2048, MMR2049, MMR2017, MMR2016, MMR2018, and MMR2012). **c** Extracts from the parental and mutant strains were analysed by HPLC. **3** represents epothilone C, **4** represents epothilone D. Statistical analysis was performed using Student’s t-test (**p < 0.01, ***p < 0.001). Error bars represent one standard deviation from three biological replicates (n = 3)

In engineering the EPOAT4 domain, both replacement of the entire module and domain swapping are possible. Considering that all chimaeric epothilone PKSs obtained through module exchange have lower product yields, we performed domain-swap experiments. We engineered EPOAT4 using a fruitful AT-swap strategy reported in 2016 by Yuzawa et al. [23]. We replaced EPOAT4 with several non-native AT domains that specifically incorporate MMCoA, including AT2, AT6, AT7, and AT8 of the epothilone PKS (EPOAT2, EPOAT6, EPOAT7, and EPOAT8), AT1 from the rapamycin PKS (RAPAT1), and AT6 of the erythromycin PKS (ERYAT6) (Fig. 2a). Non-natural hybrid EPOM4 was generated by using optimal fusion sites located in the KS-AT linker (KAL) and the non-conserved N-terminal region in the post-AT linker (PAL1) for AT domain exchanges; the highly conserved post-AT linker (PAL2) retained the original structure of EPOM4 (Additional file 1: Figure S4). It is believed that the interaction between the conserved PAL2 and the KS domain can stabilize the correct conformation of KS, which is essential for the catalysed chain extension reaction. The resulting four strains show different metabolic profiles from the parental strain 104-1. As shown in Fig. 2b, the epothilone D yields in MMR2048 (EPOAT2), MMR2049 (EPOAT6), MMR2017 (EPOAT7), and MMR2016 (EPOAT8) were 26.9 mg/l, 30.0 mg/l, 28.3 mg/l, and 29.7 mg/l, respectively, as determined by HPLC analysis (Fig. 2c; Additional file 1: Figure S5). To our surprise, these four mutants still produced epothilone C. One possible explanation is that these four EPOAT domains could incorporate MCoA in their natural context, since a previous study showed that the natural producer myxobacterium *S. cellulosum* produced minor congeners harbouring MCoA at C16, C8, C6, or C4 of the epothilone structure [35]. Then, we compared the incorporation of two alternative substrates by EPOAT2, EPOAT6, EPOAT7 and EPOAT8, and the results showed that the ratio of epothilone D to epothilone C was significantly increased to approximately 3.2:1, 4.5:1, 4.6:1 and 3.9:1, i.e., enhanced by 1.3-, 2.2-, 2.3-, and 1.8-fold, respectively. However, the production of epothilones MMR2018 (RAPAT1) and MMR2012 (ERYAT6) were at lower yields (Additional file 1: Table S2, Figure S6). MMR2018 produced less than 1 µg/l epothilone C and 17 µg/l epothilone D. MMR2012 produced trace amounts of epothilone C and D (less than 1 µg/l).

**Fig. 3:**
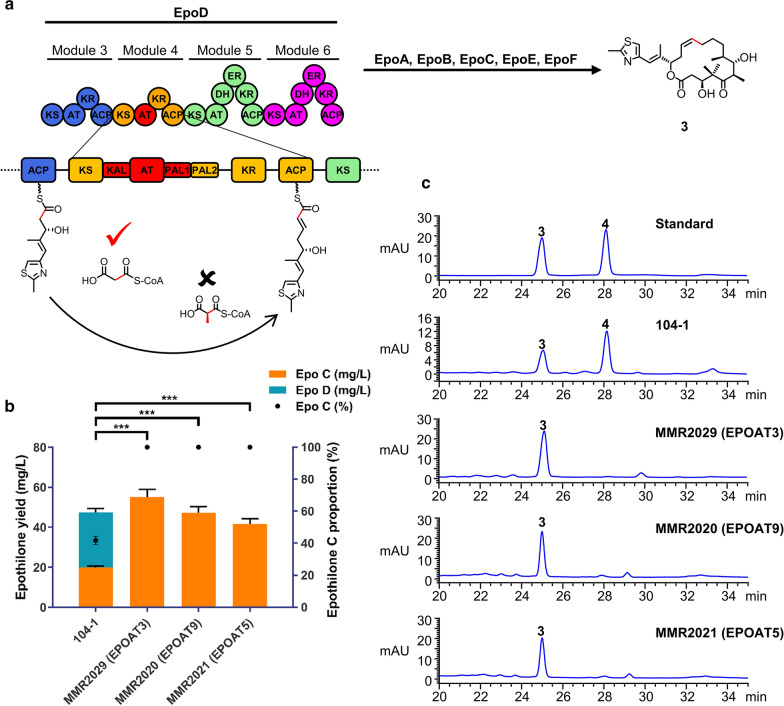
Replacing EPOAT4 with the AT domains specific to MCoA can increase the yield of epothilone C and eliminate epothilone D. **a** The module 4 AT domain of epothilone PKS (EPOAT4) was replaced with MCoA-specific AT domains (shown in red). **b** Epothilone production and epothilone C proportion from three engineered EPOAT4-swapped mutant strains (MMR2029, MMR2020, and MMR2021). **c** Extracts from the parental and mutant strains were analysed by HPLC. **3** represents epothilone C, **4** represents epothilone D. Statistical analysis was performed using Student’s t-test (***p < 0.001). Error bars represent one standard deviation from three biological replicates (n = 3)

Subsequently, we replaced EPOAT4 of the epothilone PKS with three MCoA-specific AT domains derived from the same PKS, including EPOAT3, EPOAT5, and EPOAT9 (Fig. 3a). We used the same AT-swap strategy as above. Compared with the parental strain 104-1, all three mutant strains produced the desired epothilone C, while epothilone D disappeared (Fig. 3b, c; Additional file 1: Figure S5), which was consistent with the design expectations. The epothilone C yields in MMR2029 (EPOAT3), MMR2021 (EPOAT5), and MMR2020 (EPOAT9) were 55.1 mg/l, 42.5 mg/l, and 47.3 mg/l, respectively (Additional file 1: Table S2). Compared to those of the parental strain 104-1, the titres of epothilone C increased by approximately 1.8-, 1.1- and 1.4-fold, respectively.

### Site-directed mutagenesis confirms that Ser310 in the HASH motif is the key residue for MMCoA recognition by EPOAT4

**Fig. 4:**
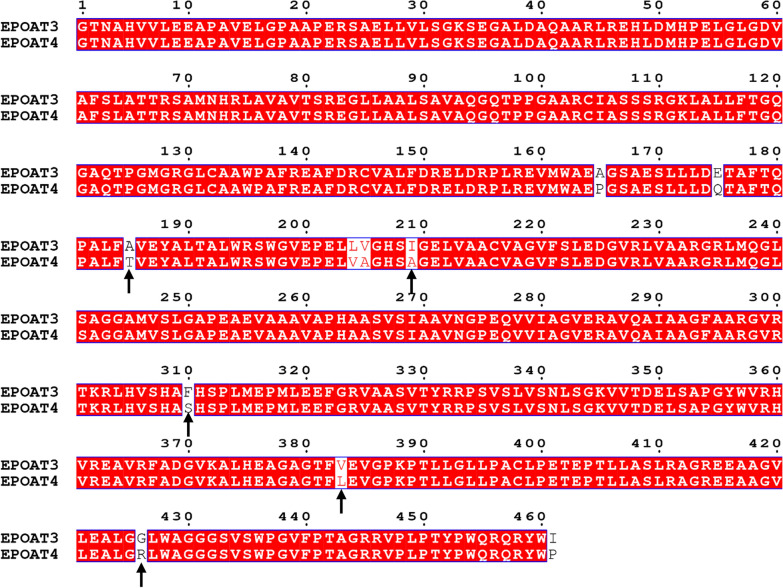
Sequence alignment of EPOAT3 and EPOAT4. The residues in the EPOAT3 domain from module 4 of the MMR2029 mutant that are labelled by black arrows were mutated to the corresponding residues of the EPOAT4 domain

**Fig. 5:**
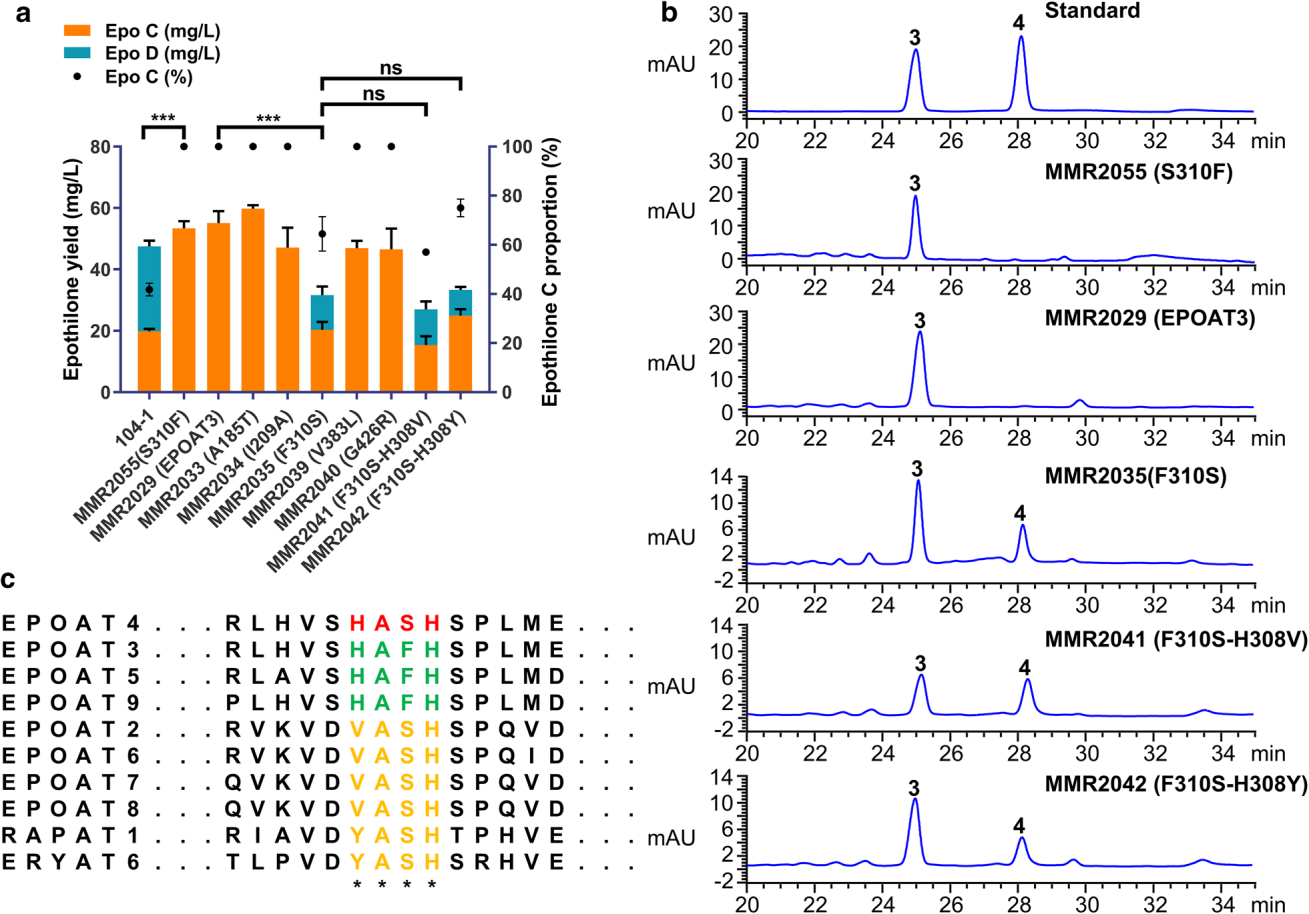
Site-directed mutagenesis of the AT domain. **a** Production and distributions of epothilones in parental and mutant strains. **b** Extracts from the parental and site-directed mutant strains were analysed by HPLC. **3** represents epothilone C, **4** represents epothilone D. **c** Identification of residues that are specifically related to AT domain substrates through protein sequence alignments. Statistical analysis was performed using Student’s t-test (***p < 0.001, * ns* not significant). Error bars represent one standard deviation from three biological replicates (n = 3)

We conducted site-directed mutagenesis of the AT domain to investigate which residues are key to changing the substrate selectivity of this domain. The above result indicated that the AT-swap strain MMR2029 (EPOAT3) produced only epothilone C (Fig. 3b, c), illustrating the exclusive substrate specificity of EPOAT3 for MCoA. Based on amino acid sequence alignments of EPOAT3 (selecting MCoA) and EPOAT4 (selecting MMCoA or MCoA), we found that these two ATs differ in only nine amino acids but possess different substrate specificities (Fig. 4). Therefore, we speculated that some of these nine amino acids in EPOAT4 are correlated with MMCoA selectivity. After further sequence alignments of these nine amino acids in ten AT domains, we excluded four amino acids in EPOAT4: Pro165, Gln175, Val204, and Ala205. Pro165 and Gln175 are identical in three domains (EPOAT4, EPOAT5 and EPOAT9), while Val204 and Ala205 are identical in EPOAT4 and EPOAT9 (Additional file 1: Table S3). The above result also showed that EPOAT5 and EPOAT9 exclusively recognize MCoA (Fig. 3b, c), so we inferred that these four amino acids are not the reason for the EPOAT4 domain recognition of MMCoA. Then, we identified the remaining five residues (Thr185, Ala209, Ser310, Leu383, and Arg426) in the EPOAT4 domain. We individually mutated five residues from the MCoA-specific EPOAT3 domain in module 4 of the MMR2029 mutant to the corresponding residues of the MMCoA-specific EPOAT4 domain to give MMR2033 (A185T), MMR2034 (I209A), MMR2035 (F310S), MMR2039 (V383L), and MMR2040 (G426R). Compared to MMR2029 (which produced only epothilone C), in these five variants, only mutation F310S resulted in a promiscuous AT domain that incorporated both extender units, and the proportion of epothilone D varied from 0 to 36 % (Fig. 5a, b; Additional file 1: Table S2, Figure S7). These results indicate that among the residues that are different in EPOAT3 and EPOAT4, Ser310 is responsible for the MMCoA selectivity of EPOAT4. To verify this conclusion, we mutated Ser310 from the EPOAT4 of the parental strain 104-1 to phenylalanine and resulting MMR2055 (S310F). Expectedly, mutation S310F resulted in an exclusive substrate specificity of EPOAT4 for MCoA and MMR2055 (S310F) produced 50.3 mg/l epothilone C (Fig. 5a, b).

Phe310 and Ser310 are located in the HAFH motif and the YASH motif, respectively, and according to previous studies, they are related to MCoA and MMCoA specificity, respectively [36]. This motif shows divergent sequences in EPOAT3 (HAFH), ERYAT6/RAPAT1 (YASH), EPOAT7 (VASH), and EPOAT4 (HASH) domains (Fig. 5c). It thus seemed reasonable that we could alter AT selectivity to cause predominant production of epothilone D by further mutating the hybrid motif to VASH or YASH (Fig. 5c). We mutated His308 from the hybrid HASH motif in MMR3035 (F310S) to the basic residue valine or tyrosine, resulting in MMR2041 (F310S-H308V) and MMR2042 (F310S-H308Y). As shown in Fig. 5a, b, the percentage of epothilone D increased from 36 to 43 % in MMR2041. However, to our surprise, the percentage of epothilone D decreased to 25 % in MMR2042. The total yields of MMR2035, MMR2041, and MMR2042 declined somewhat compared to MMR2029 (Additional file 1: Table S2). These results showed that His308 plays an auxiliary role in the substrate recognition of EPOAT4.

### Retention and linkage of ΨKR with the KR domain are essential to obtain a productive epothilone PKS while deleting the DH and ER domains

**Fig. 6:**
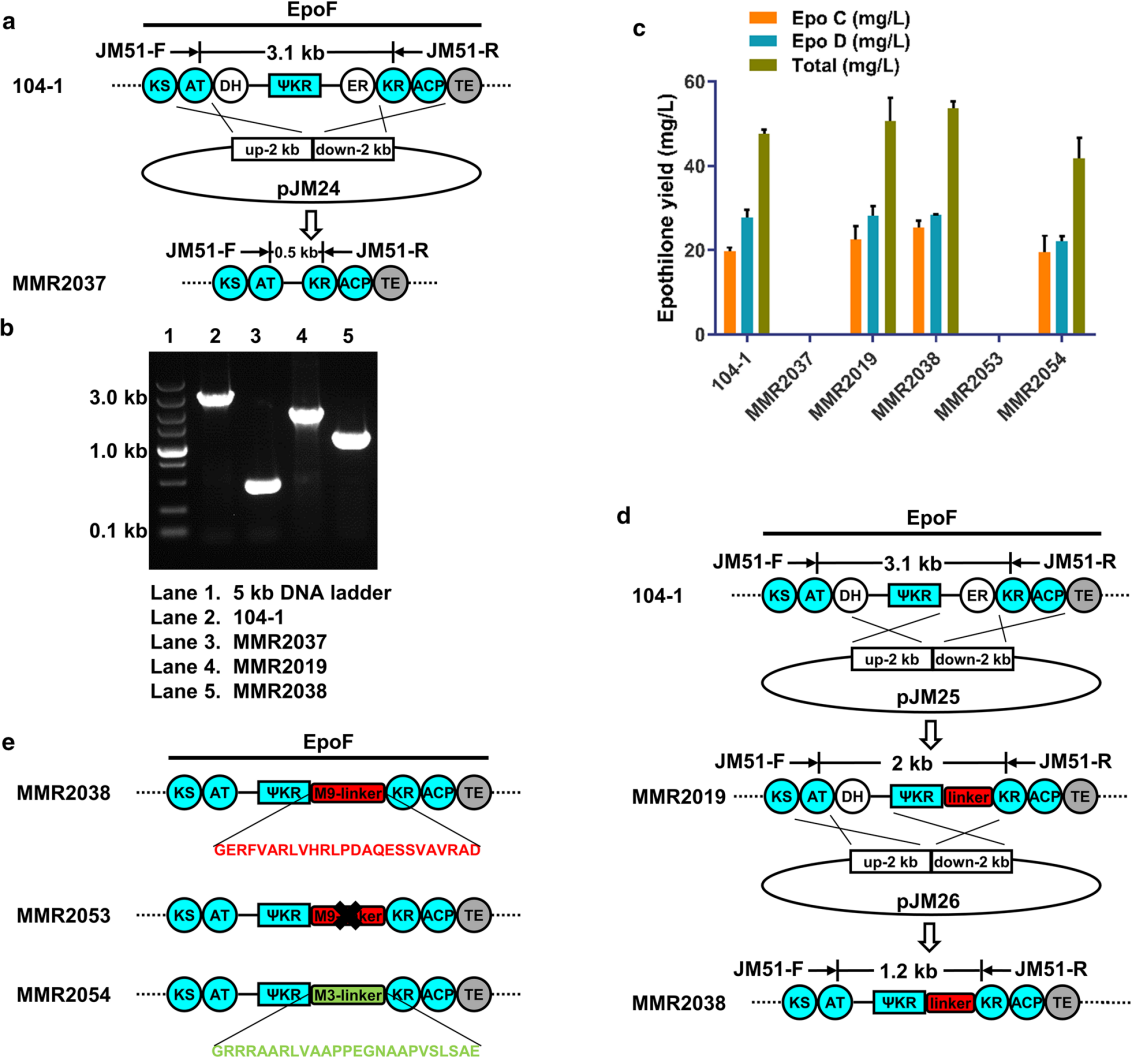
Generation of productive epothilone PKSs through deletion of the inactive module 9 DH and ER domains and fusion of the ΨKR/KR domains. **a** Scheme for DH9-ΨKR9-ER9 deletion using pJM24. **b** PCR analysis with genomic DNA from 104-1, MMR2037, MMR2019 and MMR2038 using the primers JM51-F/R. **c** Production of epothilones. **d** Schemes for ER9 deletion using pJM25 and DH9 deletion using pJM26. The ~ 25-residue ΨKR9-KR9-linker is shown in red. **e** Diagram for ΨKR9-KR9-linker deletion and replacement. The ~ 25-residue ΨKR3-KR3-linker (EPOM3) is shown in green. Error bars represent one standard deviation from three biological replicates (n = 3)

It can be deduced from the epothilone structures that the redundant EPODH9 and EPOER9 are not required. First, the DH9-ΨKR9-ER9 domains were deleted in 104-1 to generate MMR2037, and epothilone production was abolished entirely in this recombinant strain (Fig. 6a, b, c). We speculated that the inactivation of this epothilone PKS may be caused by the deletion of ΨKR, since previous studies indicate that structural ΨKR could support the function of the catalytic KR, although ΨKR has lost the ability to bind NADPH due to the extensive truncation of its core [11, 37]. Thus, we assumed that deleting DH9 and ER9 without affecting the structural integrity and activity of KR/ΨKR was a worthwhile approach to generating a productive epothilone PKS. In the follow-up experiments, we deleted the ER9 domain and DH9-ER9 didomains but retained the ΨKR9 domain between DH9 and ER9 to generate two strains, MMR2019 and MMR2038 (Fig. 6d). There were no differences in the epothilone composition compared to that of 104-1, and a small increase in total yield was detected in MMR2019 (50.6 mg/l) and MMR2038 (53.7 mg/l) (Fig. 6c; Additional file 1: Table S2). We successfully simplified the complete modifying region to a partial modifying region, and we reduced the size of the epothilone gene cluster by 1.9 kb through this method.

The efficient processivity of the modified epothilone PKS might be attributed to the use of optimal deletion sites. Amino acid sequence alignments between different modules were carried out (Additional file 1: Figure S8–S9), and when the ER9 domain was deleted in these two mutants, we retained an appropriate linker to interconnect the ΨKR9 and KR9 domains (Additional file 1: Figure S8). This modified ΨKR9-KR9-linker was the same length as the natural ΨKR-KR-linker in modules that harbour a partial modifying region (ΨKR-KR) (Additional file 1: Figure S8). To investigate the importance and flexibility of ΨKR-KR-linker, ΨKR9-KR9-linker was deleted in MMR2038 to give MMR2053 and was replaced by ~ 25-residue ΨKR3-KR3-linker from module 3 of the epothilone PKS (EPOM3) to generate MMR2054 (Fig. 6e). Predictably, the epothilone production was entirely abolished in MMR2053 (Fig. 6c), illustrating the removal of ΨKR9-KR9-linker destroyed the catalytic activity of the epothilone PKS. The model structures also showed that the ~ 25-residue ΨKR9-KR9-linker plays a vital role in maintaining the catalytic activity of the KR9 domain (Additional file 1: Figure S1b). Interestingly, the epothilone PKS in MMR2054 still has catalytic activity, and the total epothilone yields in MMR2054 were 41.8 mg/l (Fig. 6c). This consequence indicated although the ΨKR-KR-linker is indispensable, it is flexible and could be replaced by other linkers between ΨKR and KR.

## Discussion

In this study, we employed three PKS engineering strategies to produce singular metabolites, and the product distributions were altered. Compared with exchanging the whole module, domain-swap and site-directed mutagenesis methods are more effective methods. Furthermore, we successfully simplified EPOM9 by deleting inactive domains and fused ΨKR9 with the KR9 domain without decreasing the productivity of the epothilone PKS.

The activity levels of our module engineered systems were drastically diminished even though we retained appropriate covalent linkers to connect heterologous modules. We speculate that this result is attributable to the impaired specific interactions between upstream ACP (ACP_n−1_) and downstream KS (KS_n_) domains during chain translocation [7, 38, 39]. Future attempts to design productive module-exchange PKSs must pay attention to optimize the ACP_n−1_-KS_n_ interaction, as it has been suggested that mutation of the residue in ACP_n−1_, which was predicted to affect the ACP_n−1_-KS_n_ recognition, led to increased turnover [38].

In addition to exchanging the module, AT swapping and site-directed mutagenesis of AT are commonly employed strategies. Based on identification of the highly conserved AT-swap boundaries, hybrid AT-swap PKSs were constructed to produce over 1 g/l short-chain ketone *in vivo* by Yuzawa et al. [40]. This AT-swap strategy has also been successfully used in this work. However, the generation of productive AT-swap PKSs cannot be guaranteed by employing optimized AT boundaries because much lower yields of epothilones were detected in the EPOAT4-swap strains MMR2018 (RAPAT1) and MMR2012 (ERYAT6). We speculated that this result was due to the disruption of AT-ACP interactions within one module, as previous studies indicate that certain residues of ACP are responsible for cognate AT recognition during chain elongation [39, 41, 42]. The successful AT swaps in this study may be attributed to the high sequence similarity between the EPOAT4 domain and other EPOAT domains derived from the same PKS as EPOAT4; it is possible that they recognize EPOACP4 through proper AT-ACP interactions.

Site-directed mutagenesis of AT has often led to diminished turnover rates of PKSs [22, 43]; in this work, the epothilone yields of mutants that harbour mutations F310S and H308V/Y declined to a small degree compared to the starting strain. Mutation H308V had a better effect than H308Y on the MMCoA specificity of AT and resulted in an improved ratio of epothilone D. However, mutating motif HAFH to YASH/VASH alone is not enough to reverse the substrate specificity of AT domains completely. Other complementary mutations that do not destroy the catalytic activity of the complete PKS are needed. For example, using saturation mutagenesis, Sundermann et al. found the mutation Q198L also resulted in the MCoA recognition of DEBS AT6 in addition the mutations in YASH motif [44].

The failure of the deletion of the DH9-ΨKR9-ER9 region in terms of activity suggested that although ΨKR is a non-catalytic pseudo-KR domain, retention of the ΨKR domain helps generate productive PKSs. The importance of ΨKR in supporting the integrity of the catalytic KR in PKSs and mammalian fatty acid synthase (mFAS) has also been described in previous studies [11, 32, 37, 45]. We successfully fused the ΨKR9 domain with the KR9 domain by an ~ 25-residue linker in EPOM9 and maintained good catalytic activity in the KR9 domain, which is convenient for future KR9 domain engineering. Interestingly, we also found that the ΨKR-KR-linker is indispensable, but it may only play a role in connecting ΨKR and KR structurally and could be replaced by a linker of equal length from other modules.

## Conclusions

In conclusion, functional hybrid epothilone PKSs were generated, and product distributions were altered by swapping the EPOAT4 domain and by site-directed mutation of the conserved motif in AT. Furthermore, we simplified module 9 of epothilone PKS by the deletion of inactive domains without decreasing the production and proved the importance and the sequence flexibility of ΨKR-KR-linker. These results enhance our understanding of PKS domains and provide new insights into PKS engineering.

### Methods

## Chemicals, strains and culture conditions

All bacterial strains used in this study are listed in Table 1. The primers used in this study are shown in Additional file 1: Table S4 and were synthesized by Genewiz (United States). DNA sequencing was performed by Tsingke (China). The In-Fusion Cloning Kit was purchased from Novoprotein (China). DNA polymerases and a site-directed mutagenesis kit were purchased from Vazyme (China). Restriction endonucleases were purchased from Thermo Fisher Scientific (United States). The *S. brevitalea* DSM 7029 strains were cultivated at 30 °C in CYMG broth or on CYMG agar [33]. Sucrose (3 %) was added when the *sacB* gene was introduced for negative selection [46]. The *Escherichia coli* DH10B strains were grown at 37 °C in Luria-Bertani (LB) broth or on LB agar. Antibiotics were used where required at the following final concentrations: ampicillin, 100 µg/ml; spectinomycin, 50 µg/ml; apramycin, 50 µg/ml; kanamycin, 50 µg/ml; and hygromycin B, 150 µg/ml for *E. coli* and 100 µg/ml for *S. brevitalea* DSM 7029.

**Table 1:** Strains used in this study

Strains	Relevant characteristics	Source
*Escherichia coli*		
DH10B	Host strain for cloning	Invitrogen
*Saccharopolyspora erythraea*	Erythromycin-producing strain	Lab stock
*Streptomyces hygroscopicus*	Rapamycin-producing strain	Lab stock
*Schlegelella brevitalea*		
DSM 7029	Wild type	Lab stock
104-1	Epothilone producing strain	Lab stock
MMR2048	Mutant strain of 104-1 with EPOAT4 replaced by EPOAT2	This study
MMR2049	Mutant strain of 104-1 with EPOAT4 replaced by EPOAT6	This study
MMR2017	Mutant strain of 104-1 with EPOAT4 replaced by EPOAT7	This study
MMR2016	Mutant strain of 104-1 with EPOAT4 replaced by EPOAT8	This study
MMR2018	Mutant strain of 104-1 with EPOAT4 replaced by RAPAT1	This study
MMR2012	Mutant strain of 104-1 with EPOAT4 replaced by ERYAT6	This study
MMR2029	Mutant strain of 104-1 with EPOAT4 replaced by EPOAT3	This study
MMR2021	Mutant strain of 104-1 with EPOAT4 replaced by EPOAT5	This study
MMR2020	Mutant strain of 104-1 with EPOAT4 replaced by EPOAT9	This study
MMR2032	Mutant strain of MMR2029 with EPOAT3 in EPOM4 replaced by *aadA*	This study
MMR2033	Mutant strain of MMR2032 with *aadA* replaced by EPOAT3 (A185T)	This study
MMR2034	Mutant strain of MMR2032 with *aadA* replaced by EPOAT3 (I209A)	This study
MMR2035	Mutant strain of MMR2032 with *aadA* replaced by EPOAT3 (F310S)	This study
MMR2039	Mutant strain of MMR2032 with *aadA* replaced by EPOAT3 (V383L)	This study
MMR2040	Mutant strain of MMR2032 with *aadA* replaced by EPOAT3 (G426R)	This study
MMR2041	Mutant strain of MMR2032 with *aadA* replaced by EPOAT3 (F310S-H308V)	This study
MMR2042	Mutant strain of MMR2032 with *aadA* replaced by EPOAT3 (F310S-H308Y)	This study
MMR2055	Mutant strain of 104-1 with EPOAT4 (S310F)	This study
MMR2037	Mutant strain of 104-1 with EPODH9-ΨKR9-ER9 region deleted	This study
MMR2019	Mutant strain of 104-1 with EPOER9 deleted	This study
MMR2038	Mutant strain of MMR2019 with EPODH9 deleted	This study
MMR2053	Mutant strain of MMR2038 with ΨKR9-KR9-linker deleted	This study
MMR2054	Mutant strain of MMR2038 with ΨKR9-KR9-linker (EPOM9) replaced by ΨKR3-KR3-linker (EPOM3)	This study
MMR2044	Mutant strain of 104-1 with EPOM4 replaced by EPOM7	This study
MMR2024	Mutant strain of 104-1 with EPOM4 replaced by ERYM6	This study
MMR2027	Mutant strain of 104-1 with EPOM4 replaced by RAPM4	This study
MMR2026	Mutant strain of 104-1 with EPOM4 replaced by RAPM10	This study

### Plasmid and strain construction

The plasmids used in this study are shown in Additional file 1: Table S5. We discuss the method of construction of EPOAT4 replacement mutants as an example. To change the EPOAT4 domain, the recombinant plasmids pJM6-pJM14 were constructed. Briefly, using the genomic DNA of 104-1 as a template, the 2.0-kb upstream arm and 1.9-kb downstream arm were amplified using primers JM01-F/R and JM02-F/R and added to pJM1 using In-Fusion cloning to generate pJM5. The *epoAT2*, *epoAT6*, *epoAT7*, *epoAT8*, *epoAT3*, *epoAT5*, and *epoAT9* genes were cloned from the genome of *S. brevitalea* 104-1 by PCR. The *rapAT1* and *eryAT6* genes were amplified from the genomes of *Streptomyces hygroscopicus* and *Saccharopolyspora erythraea*, respectively. These fragments were ligated into pJM5 individually using In-Fusion cloning to form the recombinant plasmid pJM6-pJM14. After verification by restriction digestion analysis and sequencing, all recombinant plasmids were transformed into the parental strain *S. brevitalea* 104-1. After growing at 30 °C for 2.5 days, recombinant single-crossover colonies were selected on plates containing apramycin. We confirmed the integrated mutants by PCR and transferred the colonies into 2 ml of antibiotic-free CYMG broth at 30 °C. To force these mutants to undergo a second round of recombination, in the next three days, we transferred 10 µl of the culture into 2 ml of antibiotic-free fresh CYMG broth three times, and 1–2 % sucrose was added in each step. Then, we replicated the colonies grown on CYMG agar plates without antibiotics on CYMG agar plates with apramycin. Colonies that grew only on antibiotic-free CYMG agar plates were screened for being double-crossover mutants. The expected up-AT4swap and AT4swap-down fragments were obtained from a double crossover by PCR (Additional file 1: Figure S10a, b), and DNA sequencing further confirmed that EPOAT4 in the epothilone PKS was successfully replaced with non-native AT domains. The obtained double-crossover mutant strains were named MMR2048 (EPOAT2), MMR2049 (EPOAT6), MMR2017 (EPOAT7), MMR2016 (EPOAT8), MMR2018 (RAPAT1), MMR2012 (ERYAT6), MMR2029 (EPOAT3), MMR2021 (EPOAT5) and MMR2020 (EPOAT9).

The same method was used to construct EPOM4 replacement mutants, AT site-directed mutagenesis mutants, inactive domain deletion mutants and ΨKR9-KR9-linker deletion and replacement mutants. Plasmids pJM29-pJM32 were constructed from pJM3, and the resulting EPOM4 replacement mutants were MMR2044 (EPOM7), MMR2024 (ERYM6), MMR2027 (RAPM4), and MMR2026 (RAPM10) (Additional file 1: Figure S11). The epothilone-negative strain MMR2032 was obtained to analyse the impact of mutations by insertional inactivation via homologous recombination, where the gene encoding EPOAT3 in EPOM4 of MMR2029 was replaced by an *aadA* fragment encoding spectinomycin resistance. pJM15 was constructed from pJM4 to give MMR2032. We introduced mutation sites by site-directed mutagenesis using primers (Additional file 1: Table S4) and constructed pJM16-pJM23 from pJM5. The obtained site-directed mutagenesis strains MMR2033 (A185T), MMR2034 (I209A), MMR2035 (F310S), MMR2039 (V383L), MMR2040 (G426R), MMR2041 (F310S-H308V), MMR2042 (F310S-H308Y) and MMR2055 (S310F) were assessed by PCR, and the resulting PCR products were sequenced (Additional file 1: Figure S12). For the deletion of inactive domains, we constructed plasmid pJM24 from pJM1, pJM25 from pJM3, and pJM26 from pJM2. The resulting mutants MMR2037, MMR2019, and MMR2038 were amplified by PCR using the primers JM51-F/R to verify removal (Fig. 6b; Additional file 1: Table S4). For the deletion and replacement of ΨKR9-KR9-linker, we constructed plasmid pJM27 from pJM1, pJM28 from pJM27. The resulting mutants MMR2053 and MMR2054 were assessed by PCR and the resulting PCR products were sequenced (Additional file 1: Figure S13).

### Fermentation, extraction and HPLC analysis of epothilone production

A single colony was inoculated into 2 ml of CYMG broth supplemented with the appropriate antibiotic and then incubated at 30 °C and 220 rpm for 24 h. Then, a 250-ml conical flask harbouring 30 ml of CYMG broth and 300 µl of overnight culture was incubated with shaking for 24 h. Flask-scale fermentations were performed at 30 ℃ for 6 days in a 2-l shake flask with a 500-ml working volume. The fermentation medium (500 ml) in the 2-l shake flask was inoculated with 25 ml of preculture. The fermentation medium and extraction methods were described in our previous work [34]. We filtered the eluate by using 0.22-µm organic filter membranes and diluted the extract by adding 100 µl of extract to 900 µl of ethyl acetate. The supernatant was analysed by HPLC using an Ultimate XB-C18 column (4.6 × 250 mm, 5 μm, Welch, Shanghai, China), and its absorbance was measured at 249 nm on a 1260 Infinity instrument (Agilent Technologies, United States) after centrifugation at 12,500 rpm for 15 min. The flow rate was 1.0 ml/min, and gradient elution was performed using mobile phase solvent C (water) and solvent D (methanol). The gradient conditions were as follows: 0–5 min, hold at 65 % D; 5–15 min, linear increase from 65 to 75 % D; 15–25 min, linear increase from 75 to 80 % D; and 25–40 min, hold at 80 %. The injection volume was 10 µl.

### LC–MS analysis of epothilone production

LC–MS analysis of epothilone production was conducted using the method described in our previous study [33]. The mass values were *m/z* = 478.4 and 290.2 for epothilone C, 492.4 and 304.2 for epothilone D. A dilution series of epothilone standards was created to generate a concentration standard curve for quantification (Additional file 1: Figure S14). All samples were diluted to proper concentrations within the standard curve before detection.

### Multiple sequence alignment and structural modelling

The amino acid sequences were aligned using ClustalW (https://www.genome.jp/tools-bin/clustalw), and the alignments were produced using ESPript 3.0 (http://espript.ibcp.fr/ESPript/cgi-bin/ESPript.cgi ).

Phyre2 (http://www.sbg.bio.ic.ac.uk/phyre2/html/page.cgi?id=index) was used to generate all tertiary structure models [47]. Structure alignment of the two models was conducted by PyMOL 2.3.2.

## Supplementary Information

**Additional file 1: Table S1.** Engineered module-swap fusion sites of hybrid epothilone PKSs. **Table S2.** Yields of epothilones in mutants of *Schlegelella brevitalea* DSM 7029. **Table S3.** Alignment of nine amino acids that differ in EPOAT3 and EPOAT4. **Table S4.** Primers used for the construction of plasmids and verification of mutant strains. **Table S5.** Plasmids used in this study. **Figure S1.** Structural models of reducing domains in wild-type and mutant EPOM9s. **Figure S2.** Engineering the epothilone PKS by whole-module swapping. **Figure S3.** Extracted ion chromatogram (EIC) of LC–MS analyses of epothilone production in EPOM4-swap mutants. **Figure S4.** Multiple sequence alignment of AT domains used in this study. **Figure S5.** Extracted ion chromatogram (EIC) of LC–MS analyses of epothilone production in some AT-swap mutants to further confirm the production of epothilones. **Figure S6.** Extracted ion chromatogram (EIC) of LC–MS analyses of epothilone production in AT-swap mutants MMR2012 and MMR2018. **Figure S7.** HPLC analysis of the extracts from the parental and site-directed mutant strains. **Figure S8.** Junctions for ER domain deletion constructs. **Figure S9.** Junctions for DH domain deletion constructs. **Figure S10.** PCR analysis for the confirmation of AT-swap mutants. **Figure S11.** PCR analysis for the confirmation of module-exchange mutants. **Figure S12.** DNA sequences of the PCR products of site-directed mutants. **Figure S13.** DNA sequences of the PCR products of ΨKR9-KR9-linker deletion mutant (MMR2053) and replacement mutant (MMR2054). **Figure S14.** Standard curves calculated based on the peak areas of epothilone standards of different concentrations.

## Data Availability

All data generated or analysed during this study are included in this published article and its supplementary information files.

## Abbreviations

PKs: Polyketides
NRPs: Nonribosomal peptides
PKSs: Polyketide synthases
NRPSs: Nonribosomal peptide synthetases
MCoA: Malonyl-CoA
MMCoA: Methylmalonyl-CoA
KS: Ketosynthase
AT: Acyltransferase
ACP: Acyl carrier protein
ΨKR/KR: Ketoreductase
DH: Dehydratase
ER: Enoylreductase
TE: Thioesterase
KAL: KS-AT linker
PAL: Post-AT linker
