# Supplementary material for: Engineering the acyltransferase domain of epothilone polyketide synthase to alter the substrate specificity
Source: Microb Cell Fact. 2021 Apr 21;20:86. doi: 10.1186/s12934-021-01578-3 (PMC8058987; doi:10.1186/s12934-021-01578-3)
Supplement: Supplementary file 1 — Additional file 1: Table S1. Engineered module-swap fusion sites of hybrid epothilone PKSs. Table S2. Yields of epothilones in mutants of Schlegelella brevitalea DSM 7029. Table S3. Alignment of nine amino acids that differ in EPOAT3 and EPOAT4. Table S4. Primers used for the construction of plasmids and verification of mutant strains. Table S5. Plasmids used in this study. Figure S1. Structural models of reducing domains in wild-type and mutant EPOM9s. Figure S2. Engineering the epothilone PKS by whole-module swapping. Figure S3. Extracted ion chromatogram (EIC) of LC–MS analyses of epothilone production in EPOM4-swap mutants. Figure S4. Multiple sequence alignment of AT domains used in this study. Figure S5. Extracted ion chromatogram (EIC) of LC–MS analyses of epothilone production in some AT-swap mutants to further confirm the production of epothilones. Figure S6. Extracted ion chromatogram (EIC) of LC–MS analyses of epothilone production in AT-swap mutants MMR2012 and MMR2018. Figure S7. HPLC analysis of the extracts from the parental and site-directed mutant strains. Figure S8. Junctions for ER domain deletion constructs. Figure S9. Junctions for DH domain deletion constructs. Figure S10. PCR analysis for the confirmation of AT-swap mutants. Figure S11. PCR analysis for the confirmation of module-exchange mutants. Figure S12. DNA sequences of the PCR products of site-directed mutants. Figure S13. DNA sequences of the PCR products of ΨKR9-KR9-linker deletion mutant (MMR2053) and replacement mutant (MMR2054). Figure S14. Standard curves calculated based on the peak areas of epothilone standards of different concentrations. [file 12934_2021_1578_MOESM1_ESM.pdf]

Engineering the acyltransferase domain of epothilone polyketide synthase to alter the substrate specificity

[Huimin Wang][1] [Junheng Liang][1] [Qianwen Yue][1] [Long Li][2] [Yan Shi][1] [Guosong Chen][2] [Yue-zhong Li][3] [Xiaoying Bian][3] [Youming Zhang][3] [Guoping Zhao][1, 4] [Xiaoming Ding][1*]

[1][Collaborative Innovation Center for Genetics and Development, State Key Laboratory of Genetic Engineering, Shanghai Engineering Research Center of Industrial Microorganisms, Department of Microbiology, School of Life Sciences, Fudan University, Shanghai, People's Republic of China]

[2][The State Key Laboratory of Molecular Engineering of Polymers, Department of Macromolecular Science, Fudan University, Shanghai, People's Republic of China]

[3][Shandong University-Helmholtz Institute of Biotechnology, State Key Laboratory of Microbial Technology, School of Life Sciences, Shandong University, Qingdao, Shandong, People's Republic of China]

[4][CAS Key Laboratory of Synthetic Biology, Institute of Plant Physiology and Ecology, Shanghai Institutes for Biological Sciences, Chinese Academy of Sciences, Shanghai, People's Republic of China]

***Correspondence:**

Xiaoming Ding: Department of Microbiology, School of Life Sciences, Fudan University, Shanghai, 200438, People's Republic of China.

E-mail: [xmding74@fudan.edu.cn]

Table S1. Engineered module-swap fusion sites of hybrid epothilone PKSs. (EPOM3 = blue, intermodular linker between EPOM3 and EPOM4 = blue, EPOM7 = pink, intermodular linker between EPOM7 and EPOM8 = pink, ERYM6 = red, RAPM4 = sky blue, RAPM10 = deep pink, intermodular linker between EPOM4 and EPOM5 = orange, EPOM5 = light green)

MMR2044<br>(EPOM7)	......RGFAEQGLDSLMAVEIRKRLQGELGMPLSATLAFDHPTVERLVE<br>YLLSQALELQDRTDVRSARLPATEEPIAIVGIGCRFPGGAGTPEAFW<br>ELLDDGRDAIRPLEERWALVGVDPGDDVPRWAGLLTEAIDGFDAAF<br>FGIAPREARSLDPQHRLLLEVAWEGFEDAGIPPRSLVGSRTGVFVGV<br>CATEYLHAAVAHQPREERDAYSTTGNMLSIAAGRLSYTLGLQGPCL<br>TVDTACSSSLVAIHLACRSLRARESDLALAGGVNMLLSPDTMRALA<br>RTQALSPNGRCQTFDASANGFVRGEGCGLIVLKRLSDARRDGDRIW<br>ALIRGSAINQDGRSTGLTAPNVLAQGALLREALRNAGVEAEAIGYIE<br>THGAATSLGDPIEIEALRAVVGPARADGARCVLGAVKTNLGHLEGA<br>AGVAGLIKATLSLHHERIPRNLNFRTLNPRIRIEGTALELATEPVPWPR<br>TGRTRFAGVSSFGMSGTNAHVVLEEAPAVEPEAAAPERAAELFVLS<br>AKSVAALDAQAARLRDHLEKHVELGLGDVAFSLTTTRSAMEHRLA<br>VAASSREALRGALSAAAQGHTPPGAVRGRASGGSAPKVVFVFPGQ<br>GSQWVGMGRKLMAEEPVFRAALEGCDRAIEAEAGWSLLGELSAD<br>EAASQLGRIDVVQPVLFAMEVALSALWRSWGVEPEAVVGHSMGEV<br>AAAHVAGALSLEDAVAIICRRSRLLRRISGQGEMALVELSLEEAEAA<br>LRGHEGRLSVAVSNSPRSTVLAGEPAALSEVLAALTAKGVFWRQVK<br>VDVASHSPQVDPLREELVAALGAIRPRAAAVPMRSTVTGGVIAGPEL<br>GASYWAGNLRQPVRFAAAARALLEGGPTLFIEMSPHPILVPPLDEIQ<br>TAVEQGGAAVGSLRRGQDERATLLEALGTLWASGYPVSWARLFPAG<br>GRRVPLPTYPWQHERCWIEVEPEARRLAAADPTKDWFYRTDWPEV<br>PRAAPKSETAHGSWLLLADRGGVGEAVAAALSTRGLSCTVLHASA<br>DASTVAEQVSEAASRRNDWQGVLYLWGLDAVVDAGASADDVSEA<br>TRRATAPVLGLVRFLSAAPHPPRFWVVTRGACTVGGEPEVSLCQAA<br>LWGLARVVALEHPAAWGGLVDLDPQKSPTEIEPLVAELLSPDAEDQL<br>AFRSGRRHAARLVAAPPEGDVAPISLSAEGSYLVTGGLGGLGLLVAR<br>WLVERGARHLVLTSRHGLPERQASGGEQPPEARARIAAVEGLEAQG<br>ARVTVAAVDVAEADPMTALLAAIEPPLRGVVHAAGVFPVRPLAETD<br>EALLESVLRPKVAGSWLLHRLLRDRPLDLFVLFSSGAAVWGGKGQ<br>GAYAAANAFLDGLAHHRRARSLPALSLAWGLWAEGGMVDAKAHA<br>RLSDIGVLPMATGPALSALERLVKTSAVQRSVTRMDWTRFAPVYAA<br>RGRRNLLSALVAEDERTASPPVPTANRIWRGLSVAESRSALYELVRGI<br>AARVLGFADPGALDVGRGFAEQGLDSLMALEIRNRLQRELGERLSA<br>TLAFDHPTVERLVAHLLTDVLKLEDRSDTRHIRSVAADEPIAIVGAA<br>CRFPGGVEDLESYWQLLAEGVVVSAEVPADRWDAADWYDPD……
MMR2024<br>(ERYM6)	......RGFAEQGLDSLMAVEIRKRLQGELGMPLSATLAFDHPTVERLVE<br>YLLSQALELQDRTDVRSARLPATEDPIAIVGMACRFPGGVHNPGEL<br>WEFIVGGGDAVTEMPTDRGWDLDALFDPDPQRHGTSYSRHGAFLD

	GAADFDAAFFGISPREALAMDPQQRQVLETTWELFENAGIDPHSLR<br>GSDTGVFLGAAYQGYGQDAVVPEDSEGYLLTGNSSAVVSGRVAYVL<br>GLEGPAVTVDTACSSSLVALHSACGSLRDGDCGLAVAGGVSVMAGP<br>EVFTEFSRQGGLAVDGRCKAFSAEADGFGFAEGVAVVLLQRLSDAR<br>RAGRQVLGVVAGSAINQDGASNGLAAPSGVAQQRVIRKAWARAGI<br>TGADVAVVEAHGTGTRLGDPVEASALLATYGKSRGSSGPVLLGSVK<br>SNIGHAQAAAGVAGVIKVVLGLNRGLVPPMLCRGERSPLIEWSSGG<br>VELAEAVSPWPPAADGVRRAGVSAFGVSGTNAHVIIAEPPEPEPLPE<br>PGPVGVLAAANSVPVLLSARTETALAAQARLLESAVDDSVPLTALA<br>SALATGRAHLPRRAALLAGDHEQLRGQLRAVAEGVAAPGATTGTAS<br>AGGVVFVFPGQGAQWEGMARGLLSVPVFAESIAECDAVLSEVAGFS<br>ASEVLEQRPDAPSLERVDVVQPVLFSVMVSLARLWGACGVSPSAVI<br>GHSQGEIAAAVVAGVLSLEDGVRVVALRAKALRALAGKGGMVSLA<br>APGERARALIAPWEDRISVAAVNSPSSVVVSGDPEALAELVARCEDE<br>GVRAKTLPVDYASHSRHVEEIRETILADLDGISARRAAIPLYSTLHGE<br>RRDGADMGPRYWYDNLRSQVRFDEAVSAAVADGHATFVEMSPHP<br>VLTAAVQEIAADAVAIGSLHRDTAEEHLIAELARAHVHGVAVDWRN<br>VFPAAPPVALPNYPFEPQRYWLAPEVSDQLADSRYRVDWRPLATTP<br>VDLEGGFLVHGSAPESLTSAVEKAGGRVVPVASADREALAAALREV<br>PGEVAGVLSVHTGAATHLALHQSLGEAGVRAPLWLVTSRAVALGES<br>EPVDPEQAMVWGLGRVMGLETPERWGGLVDLPAEPAPGDGEAFVA<br>CLGADGHEDQVAIRDHARYGRRLVRAPLGTRESSWEPAGTALVTGG<br>TGALGGHVARHLARCGVEDLVLVSRRGVDAPGAAELEAELVALGA<br>KTTITACDVADREQLSKLLEELRGQGRPVRTVVHTAGVPESRPLHEI<br>GELESVCAAKVTGARLLDELCPDAETFVLFSSGAGVWGSANLGAY<br>SAANAYLDALAHRRRAEGRAATSVAWGAWAGEGMATGDLEGLTR<br>RGLRPMAPERAIRALHQALDNGDTCVSIADVDWERFAVGFTAARPR<br>PLLDELVTPAVGAVPAVQAAPAREMTSQELLEFTHSHVAAILGHSSPD<br>AVGQDQPFTELGFDSLTAVGLRNQLQQATGLALPATLVFEHPTVRRL<br>ADHIGQQLLKLEDRSDTQHVWSLASDEPIAIVGAACRFPGGVEDLE<br>SYWQLLAEGVVVSAEVPADRWDAADWYDPD……
MMR2027<br>(RAPM4)	......RGFAEQGLDSLMAVEIRKRLQGELGMPLSATLAFDHPTVERLVE<br>YLLSQALELQDRTDVRSARLPATEEPLAIVGMACRLPGGVSSPEDL<br>WRLVESGTDAVSGFPTDRGWDVENLYDSDPEAAGKSYCVQGGFLD<br>TAAGFDAGFFGISPREALAMDPQQRLLLEVSWEAFERAGIEPGSVR<br>GSDTGVFIGAFPVGYGAGFDREGYGATSGPSVLSGRVSYVFGLEGP<br>AITMDTACSSSLVALHLAAQALRNGECSMALAGGVTVMATPEVFTE<br>FARQRGLASDGRCKAFADSADGAGFSEGAGLLLVERLSDARRNGH<br>QVLAVVRGSAVNQDGASNGLTAPNGPSQQRVIRAALSNAGLSTADV<br>DVVEAHGTGTTLGDPIEAQALLATYGQDREQPLLLGSLKSNIGHTQ<br>AASGVSGVIKMVMALRHGFVPRTLHVDEPSRHVDWAAGAVELVRE<br>NQPWPGTDRPRRAGVSSFGVSGTNAHVVLESAPPAQPAEEEQPVET<br>PVVASDVLPLVISAKTQPALTEHEDRLRAYLAASPGADTRAVASTLA<br>VTRSVFEHRAVLLGDDAVTGTAVTDPRVVFVFPGQGWQWLGMGSA

	LRDSSVVFAERMAECAAALSEFVDWDLFAVLDDPAVVDRVDVVQP<br>ASWAVMVSLAAVWQAAGVRPDAVIGHSQGEIAAACVAGAVSLRDA<br>ARIVTLRSQAIARGLAGRAAMASVALPAHEIELVDGAWIAAHNGPA<br>STVIAGTPEAVDHVLTAHEARGVRVRRITVDYASHTPHVELIRDELL<br>GITAGIGSQPPVVPWLSTVDGSWVDSPLDGEYWYRNLREPVGFHPA<br>VSQLQAQGDAVFVEVSASPVLLQAMDDDVVTVATLRRDDGDATR<br>MLTALAQAYVHGVTVDWPAILGTTTARVLDLPTYAFQHQRYWVKS<br>VDRAAADGHPLLGAVVELPESDGVLLTGRVSLATHAWLADHAVWG<br>RVLLPGTAFVELVVHAAGEVGCDVVDELVIETPLLLPQTGGVQLSV<br>SVGEADESGHRVVTVFSRADNADTWTRHVSATVRVSDTTVPPSDLT<br>AWPPAQAKPVDVAGFYDQLTGMGYEYGPAFQGLQAAWRDGDTVF<br>AEVALAEEQVREAARYAVHPALLDAALHACTLNASDAEVGVGLPFS<br>WNGVRVHAGGSAMLRVAVTQAADGWSVRVADDIGRPVASVGSLVT<br>RPVTADALGSAADDLLALTWAGIPTPQQTGLTVGRFEELVSDGDVP<br>VPEVAVFTALPDNDDDPLEQTRKLTGQVLQAVQEWLGGERFSDSTL<br>VVRTGTGLAAAAVSGWMRSAQSEHPGRFVLVESDDDALAPDQLAA<br>AVGLDEPRLRISDGRFEAPRLTRTHAAEPESEKVWDPDGTVLITGGS<br>GVLAGIAARHLVAERGVRHLLLLSRSAPDEALINQLGELGARVETA<br>ACDVSDRAALAQVLAGVSPEHPLTAVIHTAGALDDGVVESLTAQRL<br>DAVLRPKADGAWNLHELTRDADLAAFVMYSSAAGVLGSAGQANY<br>AAANAFVDALAEQRRAEGLPALAVAWGLWEDASGLTADLTDTDRD<br>RIRRGGLRAISAEYGMGLFDSASRHSEPVLVGAAMEPVRDAEVPAL<br>LRSLHRPIARRAASTGDSSVQWLAALAPEERAKALLRVVCDSAATV<br>LGHADIDSIPVTAAFKDLGVDSLTAVDLRNSLAKATGLRLPPTLVFD<br>YPTPTALAARLDELFLKLEDRSDTQHVWSLASDEPIAIVGAACRFPG<br>GVEDLESYWQLLAEGVVVSAEVPADRWDAADWYDPD……
MMR2026<br>(RAPM10)	......RGFAEQGLDSLMAVEIRKRLQGELGMPLSATLAFDHPTVERLVE<br>YLLSQALELQDRTDVRSARLPATEEPLAIVGMACRLPGGVSSPEDL<br>WRLVESGTDAISGFPTDRGWDVENLYDPDPDAPGKSYSVQGGFLD<br>AAAGFDASFFGISPREALAMDPQQRLMLEVSWEAFERAGIEPGSVR<br>GSDTGVFIGAYPGGYGIGADLGGFGTTAGAASVLSGRVSYFFGLEG<br>PAFTVDTACSSSLVALHQAGYALRQGECSLALVGGVTVMPTPQTFV<br>EFSRQRGLSADGRCKAFADAADGTGWAEGVGVLLVERLSDAQAN<br>GHQILAVVRSSAVNQDGASNGLSAPNGPSQQRVIRAALSNAGLAPH<br>EVDVVEAHGTGTTLGDPIEAQAVIATYGQGRGEPLLLGSLKSNVGH<br>TQAAAGVSGVIKMVMALQHSMVPRTLHVDEPSRHVDWSAGAVEL<br>VAENQPWPETGRPRRAGVSSFGISGTNAHVILESAPAQSVGDTAGST<br>PVLVSELVPLVISAKTQPALTEHEDRLRAYLAASPGVDIRAVASTLAV<br>TRSVFEHRAVLLGDETVTGTAVSDPRIVFVFPGQGWQWLGMGSAL<br>RDSSVVFAERMAECAAALSEFVDWDLFAVLDDPAVVDRVDVVQPA<br>SWAVMVSLAAVWQAAGVRPDAVIGHSQGEIAAACVAGAVSMRDA<br>ARIVTLRSQAIARGLAGRGAMASVALPAQDVELVDGAWIAAHNGP<br>ASTVIAGTPEAVDHVLTALRQRGAGAADHVDYASHTPHVELIRDEL<br>LDITSDSSSQDPLVPWLSTVDGTWVDSPLDGEYWYRNLREPVGFHP

	AVSQLQAQGDTVFVEVSASPVLMQAMDDDVVTVATLRRDDGDAT<br>RMLTALAQAYVHGVTVDWRAVLGDVPATRVLDLPTYAFQHQRYW<br>AEAGRSADVSAAGLDAVGHPLLGAVLAMPGSDGVMLTGRVSLATH<br>AWLADHAVRGSVLLPGTGFVELVVRAADEVACDVVDELIVEAPLLL<br>PQTGGVQLSVSVGEADESGHRAVTVFSRADSADAWVRHVSATVSV<br>SDTTVPTSDLTAWPPAQAKPVDVAGFYDQLTRAGYEYGPAFQGLQA<br>AWRDGDTVFAEVALAEEQTQDAARFAVHPAVLDAALHAGILNTPD<br>ADRDTVRLPFSWNHVQVHVTGSATLRVAMTRVADGWGVRVADDIG<br>RPVATIGSLVTRPVAADALGSAVDDLFALTWTEIPVSQQVGVTVGKF<br>EDLADGEVPMPDVVVFTALPDSGDPLAQTRRLTAEVLQAVQVWLA<br>GERFTDSTLVVRTGTGLAAAAVSGLMRSAQSEHPGRFVLVESDDDT<br>LTPDQLAATVGLDEPRLRVIDGRYEAPRLTRTGVAEPEPEGVWDPDG<br>TVLITGGSGVLAGIAARHLVAERGVRHLLLLSRSAPDEALISELAEL<br>GAAVVDTAVCDVSDRAGLARVLAGVSPDHPLTAVIHTAGVLDDGV<br>VESLTARRLDTVLRPKADGAWNLHELTRDIDLAAFVMYSSAAGVL<br>GSAGQGNYAVANAFVDALAEQRRAEGLPALALAWGLWEDASGLTA<br>KLTGTDHDRIRRSGLRTITAERGMRLFDIASRQGEPVLVATPMEPVRE<br>VEVPALLRLLHRPVARRAASTGDSSAQWLVGLAPEERAKALLKVV<br>RDSAATVLGHADARSIPATGAFKDLGVDSLTAVELRNSLTKATGLRL<br>PATMVFDYPTPADLAARLGDLMLKLEDRSDTQHVWSLASDEPIAIV<br>GAACRFPGGVEDLESYWQLLAEGVVVSAEVPADRWDAADWYDPD<br>……

Table S2. Yields of epothilones in mutants of *Schlegelella brevitalea* DSM 7029.

Strain	Epothilone yield (mg l^{-1}) [a]		
	C (**3**)	D (**4**)	total
104-1	19.85	27.67	47.52
MMR2024 (ERYM6)	0.004	0.014	0.018
MMR2027 (RAPM4)	nd	nd	nd
MMR2026 (RAPM10)	nd	nd	nd
MMR2044 (EPOM7)	0.015	0.017	0.032
MMR2048 (EPOAT2)	8.53	26.91	35.44
MMR2049 (EPOAT6)	6.68	30.04	36.72
MMR2017 (EPOAT7)	6.11	28.30	34.41
MMR2016 (EPOAT8)	7.63	29.72	37.35
MMR2018 (RAPAT1)	$<$0.001	0.017	0.017
MMR2012 (ERYAT6)	$<$0.001	$<$0.001	$<$0.001
MMR2029 (EPOAT3)	55.08	nd	55.08
MMR2020 (EPOAT9)	47.27	nd	47.27
MMR2021 (EPOAT5)	42.53	nd	42.53
MMR2033 (A185T)	59.77	nd	59.77
MMR2034 (I209A)	47.11	nd	47.11
MMR2035 (F310S)	20.34	11.26	31.60
MMR2039 (V383L)	46.90	nd	46.90
MMR2040 (G426R)	46.57	nd	46.57
MMR2041 (F310S-H308V)	15.35	11.63	26.98
MMR2042 (F310S-H308Y)	24.99	8.29	33.28
MMR2055 (S310F)	50.34	nd	50.34
MMR2037	nd	nd	nd
MMR2019	22.49	28.13	50.62
MMR2038	25.33	28.33	53.66
MMR2053	nd	nd	nd
MMR2054	19.61	22.14	41.75

nd not detected

a The yields of epothilones are averages of three biological replicates under identical cultivation conditions.

Table S3. Alignment of nine amino acids that differ in EPOAT3 and EPOAT4. Nine amino acids in an MMCoA/MCoA-specific AT domain are shown in red; nine amino acids in MCoA-specific AT domains are shown in green; nine amino acids in MMCoA-specific AT domains are shown in orange. Four amino acids (Pro165, Gln175, Val204, and Ala205) that are identical in three AT domains (EPOAT4, EPOAT5, and EPOAT9) are highlighted in yellow.

M/MM	M	M	M	MM	MM	MM	MM	MM	MM
EPOAT4	EPOAT3	EPOAT5	EPOAT9	EPOAT2	EPOAT6	EPOAT7	EPOAT8	ERYAT6	RAPAT1
Pro165	A	P	P	E	E	E	E	P	/
Gln175	E	Q	Q	R	R	R	R	R	R
Thr185	A	A	A	A	A	A	A	S	A
Val204	L	L	V	V	V	V	V	V	V
Ala205	V	L	A	I	V	V	V	I	V
Ala209	I	I	L	M	M	M	M	Q	Q
Ser310	F	F	F	S	S	S	S	S	S
Leu383	V	V	V	V	V	I	I	V	V
Arg426	G	A	G	A	A	T	T	R	Q

Table S4. Primers used for the construction of plasmids and verification of mutant strains.

Primer	Sequence (5'–3') *	Use
JM01-F	*GACTACCGGGCGGCATATCA*TGGGTGCTGCA CACCCTTAC	Amplification of upstream homologous arm for *epoAT4*
JM01-R	GCTCATCCCGAACGAGCTCA	
JM02-F	*TGAGCTCGTTCGGGATGAGC*CTGCCGACCTA TCCGTGGCA	Amplification of downstream homologous arm for *epoAT4*
JM02-R	TACATCGACGAGCAGATGCT	
JM03-F	*TGAGCTCGTTCGGGATGAGC*GGCACCAACGC CCATGTCGT	Amplification of *epoAT2*
JM03-R	CGGTACCCGCCGCCCCCCG	
JM04-F	*TGAGCTCGTTCGGGATGAGC*GGGACCAACGC GCACGTGGT	Amplification of *epoAT6*
JM04-R	CGGCACGCGACGGAGGCCCG	
JM05-F	*TGAGCTCGTTCGGGATGAGC*GGGACCAACGC GCATGTGGT	Amplification of *epoAT7*
JM05-R	CGGAACCCGCCTGCCGCCCG	
JM06-F	*TGAGCTCGTTCGGGATGAGC*GGGACCAACG CGCATGTGGT	Amplification of *epoAT8*
JM06-R	CGGAACCCGCCTGCCGCCCG	
JM07-F	*TGAGCTCGTTCGGGATGAGC*GGTACGAACG CCCACGTCAT	Amplification of *rapAT1*
JM07-R	GTCCAGCACCCGGGCTGCGG	
JM08-F	*TGAGCTCGTTCGGGATGAGC*GGGACGAACG CGCACGTGAT	Amplification of *eryAT6*
JM08-R	CGCCACCGGAGGTGCCGCCG	
JM09-F	*TGAGCTCGTTCGGGATGAGC*GGAACGAACG CGCACGTGGT	Amplification of *epoAT3*
JM09-R	CGGCACCCGCCGCCCAGCCG	
JM10-F	*TGAGCTCGTTCGGGATGAGC*GGAACCAACG TGCATGTCGT	Amplification of *epoAT5*
JM10-R	AGCCACGCGGCGCGCGCCAT	
JM11-F	*TGAGCTCGTTCGGGATGAGC*GGCACCAACG TCCATGTCGT	Amplification of *epoAT9*
JM11-R	CGGTACCCGCCGTCCGCCCG	
JM12-F	CCGAAGAGGTCGGCAAAGTC	*epoAT4* replacement verification for upstream fragment

JM12-R	CGCCTTTGGTCACGTAAGTC	*epoAT4* replacement verification for downstream fragment
JM13-R	GCCTGCAGATGATCTCGTAG	*epoAT2* verification for upstream fragment
JM13-F	ACAACCTCAGGCAGCCTGTG	*epoAT2* verification for downstream fragment
JM14-R	AGCTGCGAGGTGGTCTCATC	*epoAT6* verification for upstream fragment
JM14-F	ACGAGCGCCTGTCCATGTTG	*epoAT6* verification for downstream fragment
JM15-R	CCAAGCTCGACATGCTTCTC	*eryAT7 and epoAT8* verification for upstream fragment
JM15-F	CATGGGCCGAAAGCTCATGG	*eryAT7 and epoAT8* verification for downstream fragment
JM16-R	CCAGCTCCGAATCCACTACG	*rapAT1* verification for upstream fragment
JM16-F	GGTACCGGAACTTGCGTGAG	*rapAT1* verification for downstream fragment
JM17-R	CCAATGCCGTCAACGGAACC	*eryAT6* verification for upstream fragment
JM17-F	GCCACCTTCGTCGAGATGAG	*eryAT6* verification for downstream fragment
JM18-R	CTGTCCGGTGAACAGCAACG	*epoAT3* verification for upstream fragment
JM18-F	TCGCTGGTGAGCAACCTGAG	*epoAT3* verification for downstream fragment
JM19-R	AAGCCAGCTTACCGCGTGAG	*epoAT5* verification for upstream fragment
JM19-F	AGTATTGGGTCCGGCATGTG	*epoAT5* verification for downstream fragment
JM20-R	GAACAGGAAGGCGAGCTTGC	*epoAT9* verification for upstream fragment
JM20-F	GAAATTCGTGCAGCAGATCG	*epoAT9* verification for downstream fragment
JM21-F	*CATTGGTAACTCGAGCTGTG*GTGGTCACGGACGAGCTGAG	Amplification of upstream homologous arm for *epo-module4*
JM21-R	CTCTGTCGCCGGCAACCGAG	
JM22-F	*TCCGGTCGGTGGCGGCGGAT*GAGCCCATCGCCATCGTGGG	Amplification of downstream homologous arm for *epo-module4* (for *epo-module7*)
JM22-R	GGGATACGCACGAAGAGGCT	

JM23-F	CTGAAGCTGGAGGATCGCAG	Amplification of downstream homologous arm for *epo-module4* (for *ery-module7*, *rap-module4 and rap-module10*)
JM23-R	GGGATACGCACGAAGAGGCT	
JM24-F	*CTCGGTTGCCGGCGACAGAG*GAGCCGATCG CCATCGTGGG	Amplification of *epo-module7*
JM24-R	ATCCGCCGCCACCGACCGGA	
JM25-F	*CTCGGTTGCCGGCGACAGAG*GACCCGATCG CGATCGTCGG	Amplification of *ery-module6*
JM25-R	*CTGCGATCCTCCAGCTTCAG*GAGCTGCTGTC CTATGTGGT	
JM26-F	*CTCGGTTGCCGGCGACAGAG*GAGCCGTTGG CGATTGTGGG	Amplification of *rap-module4*
JM26-R	*CTGCGATCCTCCAGCTTCAG*GAACAACTCG TCCAGCCGGG	
JM27-F	*CTCGGTTGCCGGCGACAGAG*GAGCCGCTGG CGATCGTGGG	Amplification of *rap-module10*
JM27-R	*CTGCGATCCTCCAGCTTCAG*CATGAGGTCG CCCAGCCGGG	
JM28-F	TCGTTGTTGCTCGACGAGAC	*epo-module4* replacement verification for upstream fragment
JM28-R	CATGCCGGACCCAATACTCG	*epo-module4* replacement verification for downstream fragment
JM29-R	CGCTTGAGCACGATCAGACC	*epo-module7* verification for upstream fragment
JM29-F	TGGCTCTGGAGATCCGTAAC	*epo-module7* verification for downstream fragment
JM30-R	ACCCACACGCCGAATGCAAG	*ery-module6* verification for upstream fragment
JM30-F	CGCAGGAGTTGCTGGAGTTC	*ery-module6* verification for downstream fragment
JM31-R	CTGCACGCAATACGACTTCC	*rap-module4* verification for upstream fragment
JM31-F	CTACCGGTGACTCGTCAGTG	*rap-module4* verification for downstream fragment
JM32-R	CCTGCACGCTGTAGGACTTC	*rap-module10* verification for upstream fragment
JM32-F	CGGTGCGTTCAAGGATTTGG	*rap-module10* verification for downstream fragment

JM33-F	*GACTACCGGGCGGCATATCA*TGGGTGCTGC ACACCCTTAC	Amplification of upstream homologous arm for *epoAT3 (epo-module4)* in MMR2029
JM33-R	CTCCGCCCACATCACCTCGC	
JM34-F	*GCTAGGGAAGATGCCAGGAA*GGTGTTCTCG CTGGAAGATG	Amplification of downstream homologous arm for *epoAT3 (epo-module4)* in MMR2029
JM34-R	AGCATCTGCTCGTCGATGTA	
JM35-R	GCGAGATCACCAAGGTAGTC	*aadA* verification for upstream fragment
JM35-F	CTCCACCGCTGATGACATGC	*aadA* verification for downstream fragment
JM36-F	*ACGGCGTTCACCCAGCCCGC*GCTCTTCACG GTGGAGTACG	Targeted mutagenesis of *epoAT3 (epo-module4)* in MMR2029 to change Ala185 to Thr
JM36-R	GCGGGCTGGGTGAACGCCGT	
JM37-F	*TGGGGCGTAGAGCCGGAGCT*CCTGGTTGGG CATAGCGCCG	Targeted mutagenesis of *epoAT3 (epo-module4)* in MMR2029 to change Ile209 to Ala
JM37-R	AGCTCCGGCTCTACGCCCCA	
JM38-F	*CGTGCGCACCAAGCGGCTGC*ATGTCTCGCA CGCGTCCCAC	Targeted mutagenesis of *epoAT3 (epo-module4)* in MMR2029 to change Phe310 to Ser
JM38-R	GCAGCCGCTTGGTGCGCACG	
JM39-F	*GGACGTTCCTCGAAGTGGGC*CCGAAGCCGA CGCTGCTCGG	Targeted mutagenesis of *epoAT3 (epo-module4)* in MMR2029 to change Val383 to Leu
JM39-R	GCCCACTTCGAGGAACGTCC	
JM40-F	*GGCAGGCTGTGGGCCGGCGG*CGGCTCGGT CAGCTGGCCGG	Targeted mutagenesis of *epoAT3 (epo-module4)* in MMR2029 to change Gly426 to Arg
JM40-R	CCGCCGGCCCACAGCCTGCC	
JM41-F	*GTCTCGGTCGCGTCCCACTC*GCCGCTGATG GAACCGATGC	Targeted mutagenesis of *epoAT3 (epo-module4)* in MMR2029 to change His308 to Val and Phe310 to Ser
JM41-R	GAGTGGGACGCGACCGAGAC	
JM42-F	*GTCTCGTACGCGTCCCACTC*GCCGCTGATG GAACCGATGC	Targeted mutagenesis of *epoAT3 (epo-module4)* in MMR2029 to change His308 to Tyr and Phe310 to Ser
JM42-R	GAGTGGGACGCGTACGAGAC	
JM43-F	*GTTCCACTCGCCGCTGATGG*AACCGATGCT GGAGGAGTTC	Targeted mutagenesis of *epoAT4 (epo-module4)* in 104-1 to change Ser310 to Phe
JM43-R	CCATCAGCGGCGAGTGGAAC	
JM44-F	CCAACCTTGGGCACATGGAG	Verification for mutation of *epoAT3 (epo-module4)* in MMR2029 and mutation of *epoAT4* in 104-1
JM44-R	TCGCCTCCTCTGATTTCTGG	

JM45-F	*GACTACCGGGCGGCATATCA*ATGGCCACTG CCGGACATTC	Amplification of upstream homologous arm for *epoDH9-ΨKR9-ER9*
JM45-R	ACCCAGAAGGGGGTGGCCCC	
JM46-F	*GGGGCCACCCCCTTCTGGGT*GAATCCAGCG TCGCCGTCCG	Amplification of downstream homologous arm for *epoDH9-ΨKR9-ER9*
JM46-R	TCATTTTGCCTCGAACGCCG	
JM47-F	*GACTACCGGGCGGCATATCA*ACCAAACCGC TGCATGTCTC	Amplification of upstream homologous arm for *epoER9*
JM47-R	CTGAGCGTCGGGCAGCCGGT	
JM48-F	*ACCGGCTGCCCGACGCTCAG*GAATCCAGCG TCGCCGTCCG	Amplification of downstream homologous arm for *epoER9*
JM48-R	TCATTTTGCCTCGAACGCCG	
JM49-F	*GCATTGGTAACTCGAGCTGT*GACATGGCGA TCGGATCTGG	Amplification of upstream homologous arm for *epoDH9*
JM49-R	TCATTTTGCCTCGAACGCCG	
JM50-F	*AAAGGAATAGGGTGCTGGGA*GGAGACGCTC GCGTATTGCT	Amplification of downstream homologous arm for *epoDH9*
JM50-R	GAGTTGGACGACTCGCTGAC	
JM51-F	TGGTCGGGAGTCTTCCCTTC	Verification for inactive domains deletions of *epo-module9*
JM51-R	AGCAGCCCGTCATCCACAAG	
JM52-F	GTAGCACCTGAAGTCAGCCCACACGCCTC TAGAACCCATC	Amplification of upstream homologous arm for *ΨKR9-KR9-linker*
JM52-R	ACCGCGCAGCGCGACCTCCT	
JM53-F	AGGAGGTCGCGCTGCGCGGTGGCACCTAC CTTGTGACCGG	Amplification of downstream homologous arm for *ΨKR9-KR9-linker*
JM53-R	GGGCTGACTTCAGGTGCTAC	
JM54-F	AGGAGGTCGCGCTGCGCGGTGGGCGCCGG CGCGCAGCGCG	Amplification of *ΨKR3-KR3-linker*
JM54-R	CCGGTCACAAGGTAGGTGCCCTCCGCAGA CAGCGACACCG	
JM55-F	CAGATGTCGATGCCGATGCC	Verification for *ΨKR9-KR9-linker* deletion and replacement
JM55-R	ACAAGACCTGCCGCATGCAC	

* Homologous arms for In-Fusion cloning are in italics, and the position of point mutations leading to amino acid changes in the target proteins are highlighted in red.

Table S5. Plasmids used in this study.

**Plasmid**	**Relevant characteristics***	**Source**
pJM1	p15A_ori, *sacB*, *Apra*^{R}	Lab stock
pJM2	p15A_ori, *sacB*, *Amp*^{R}, *Hyg*^{R}	Lab stock
pJM3	p15A_ori, *sacB*, *Amp*^{R}, *Apra*^{R}	Lab stock
pJM4	p15A_ori, *sacB*, *Spec*^{R}, *Apra*^{R}	Lab stock
pJM5	pJM1 derivative containing *epoAT4* upstream and downstream fragments, *Apra*^{R}	This study
pJM6	pJM5 with *epoAT2*, *Apra*^{R}	This study
pJM7	pJM5 with *epoAT6*, *Apra*^{R}	This study
pJM8	pJM5 with *epoAT7*, *Apra*^{R}	This study
pJM9	pJM5 with *epoAT8*, *Apra*^{R}	This study
pJM10	pJM5 with *rapAT1*, *Apra*^{R}	This study
pJM11	pJM5 with *eryAT6*, *Apra*^{R}	This study
pJM12	pJM5 with *epoAT3*, *Apra*^{R}	This study
pJM13	pJM5 with *epoAT5*, *Apra*^{R}	This study
pJM14	pJM5 with *epoAT9*, *Apra*^{R}	This study
pJM15	pJM4 derivative containing *epoAT3 (epo-module4)* upstream and downstream fragments, *Spec*^{R}, *Apra*^{R}	This study
pJM16	pJM5 with *epoAT3* mutant encoding EPOAT3 (A185T), *Apra*^{R}	This study
pJM17	pJM5 with *epoAT3* mutant encoding EPOAT3 (I209A), *Apra*^{R}	This study
pJM18	pJM5 with *epoAT3* mutant encoding EPOAT3 (F310S), *Apra*^{R}	This study
pJM19	pJM5 with *epoAT3* mutant encoding EPOAT3 (V383L), *Apra*^{R}	This study
pJM20	pJM5 with *epoAT3* mutant encoding EPOAT3 (G426R), *Apra*^{R}	This study
pJM21	pJM5 with *epoAT3* mutant encoding EPOAT3 (F310S, H308V), *Apra*^{R}	This study
pJM22	pJM5 with *epoAT3* mutant encoding EPOAT3 (F310S, H308Y), *Apra*^{R}	This study
pJM23	pJM5 with *epoAT4* mutant encoding EPOAT4 (S310F), *Apra*^{R}	This study
pJM24	pJM1 derivative containing *epoDH9-ΨKR9-ER9* upstream and downstream fragments, *Apra*^{R}	This study
pJM25	pJM3 derivative containing *epoER9* upstream and downstream fragments, *Amp*^{R}, *Apra*^{R}	This study
pJM26	pJM2 derivative containing *epoDH9* upstream and downstream fragments, *Amp*^{R}, *Hyg*^{R}	This study
pJM27	pJM1 derivative containing *ΨKR9-KR9-linker* upstream and downstream fragments, *Apra*^{R}	This study
pJM28	pJM27 with *ΨKR3-KR3-linker, Apra*^{R}	This study
pJM29	pJM3 derivative containing *epo-module7* together with *epo-module4* upstream and downstream fragments, *Amp*^{R}, *Apra*^{R}	This study
pJM30	pJM3 derivative containing *ery-module6* together with *epo-module4* upstream and downstream fragments, *Amp*^{R}, *Apra*^{R}	This study

pJM31	pJM3 derivative containing *rap-module4* together with *epo-module4* upstream and downstream fragments, Amp^R, $Apra^R$	This study
pJM32	pJM3 derivative containing *rap-module10* together with *epo-module4* upstream and downstream fragments, Amp^R, $Apra^R$	This study

* Amp^R ampicillin resistance, $Apra^R$ apramycin resistance, Hyg^R hygromycin resistance, $Spec^R$ spectinomycin resistance

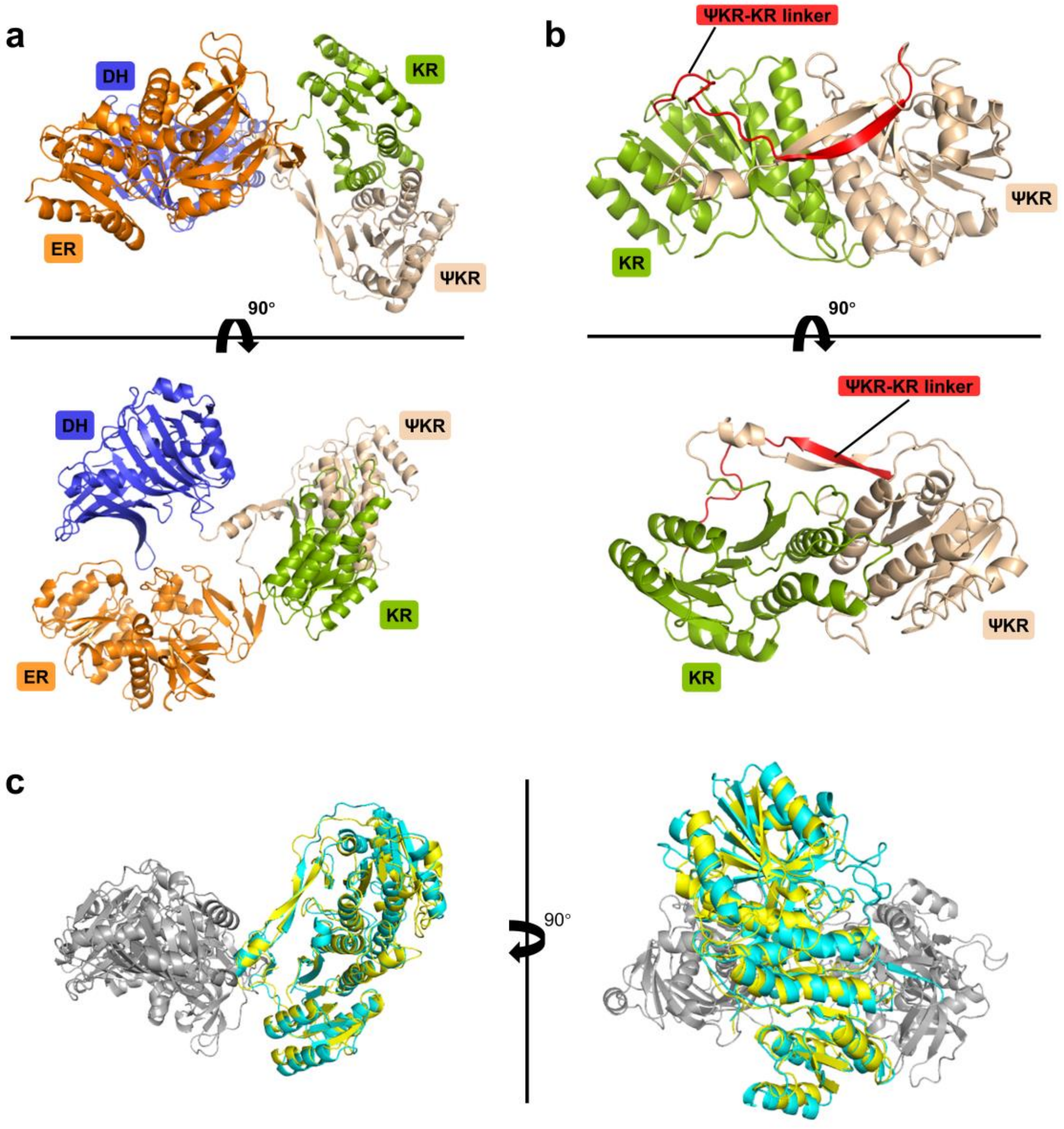

Figure S1. Structural models of reducing domains in wild-type and mutant EPOM9s. **a** Structural models of DH9-ΨKR9-ER9-KR9 domains of the wild-type EPOM9. **b** Structural models of the ΨKR9-KR9 didomain of the mutant EPOM9 (deleted DH9 and ER9 domains). **c** ΨKR9-KR9 structural model (shown in yellow) superimposed on the DH9-ΨKR9-ER9-KR9 model (shown in blue); the calculated RMSD = 1.980, which shows these two models are similar.

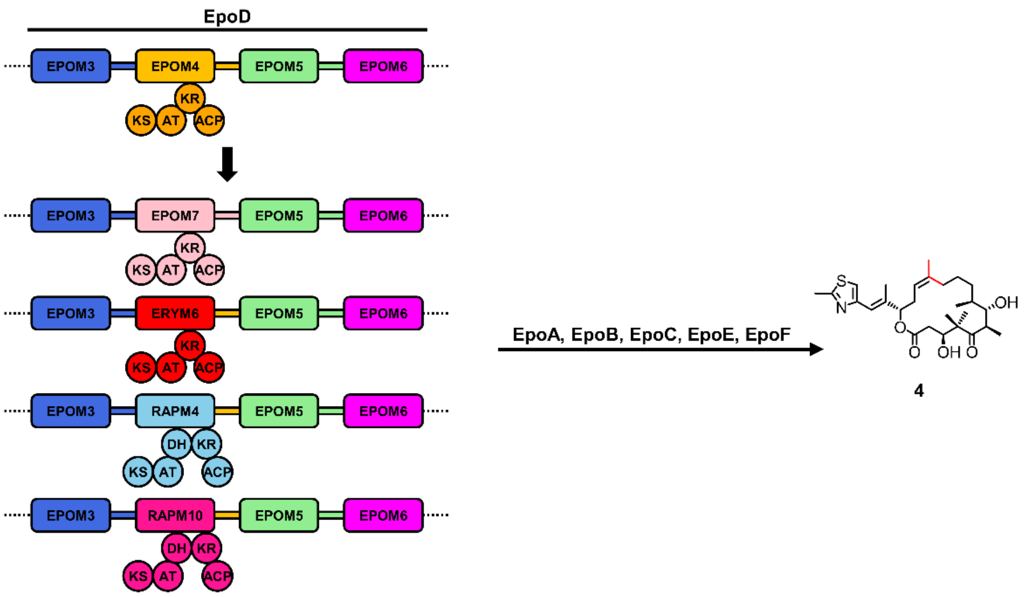

Figure S2. Engineering the epothilone PKS by whole-module swapping. The EPOM4 was replaced with EPOM7 while the natural intermodular linker between EPOM3 and EPOM4 (shown in blue) and the linker between EPOM7 and EPOM8 (shown in pink) were retained. EPOM4 was replaced with ERYM6, RAPM4, or RAPM10 while the natural intermodular linker between EPOM3 and EPOM4 (shown in blue) and the linker between EPOM4 and EPOM5 (shown in orange) were retained.

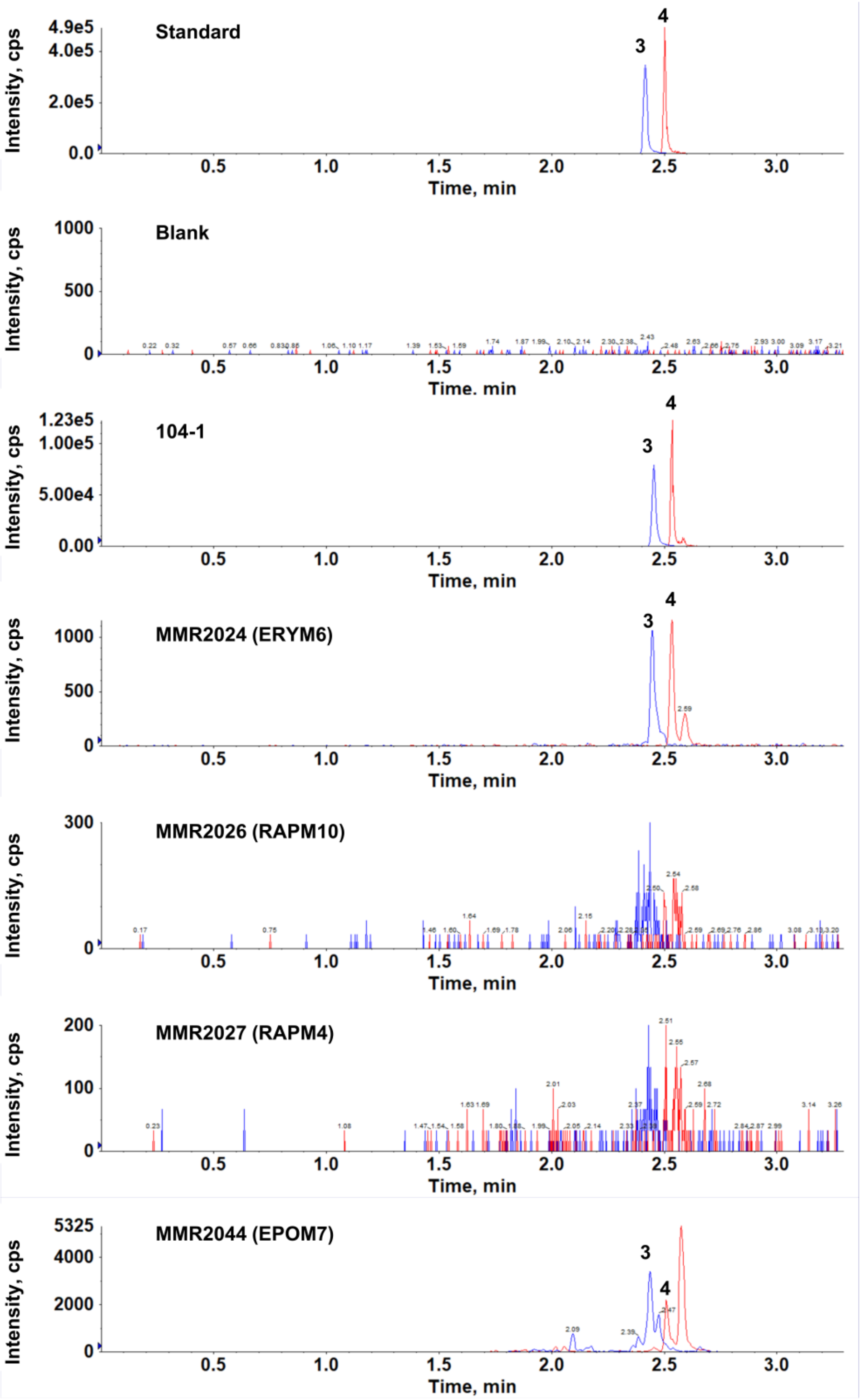

Figure S3. Extracted ion chromatogram (EIC) of LC–MS analyses of epothilone production in EPOM4-swap mutants. Peaks corresponding to epothilone C (selected for *m/z* = 478.4 and 290.2), epothilone D (selected for *m/z* = 492.4 and 304.2) were shown in blue and red, respectively.

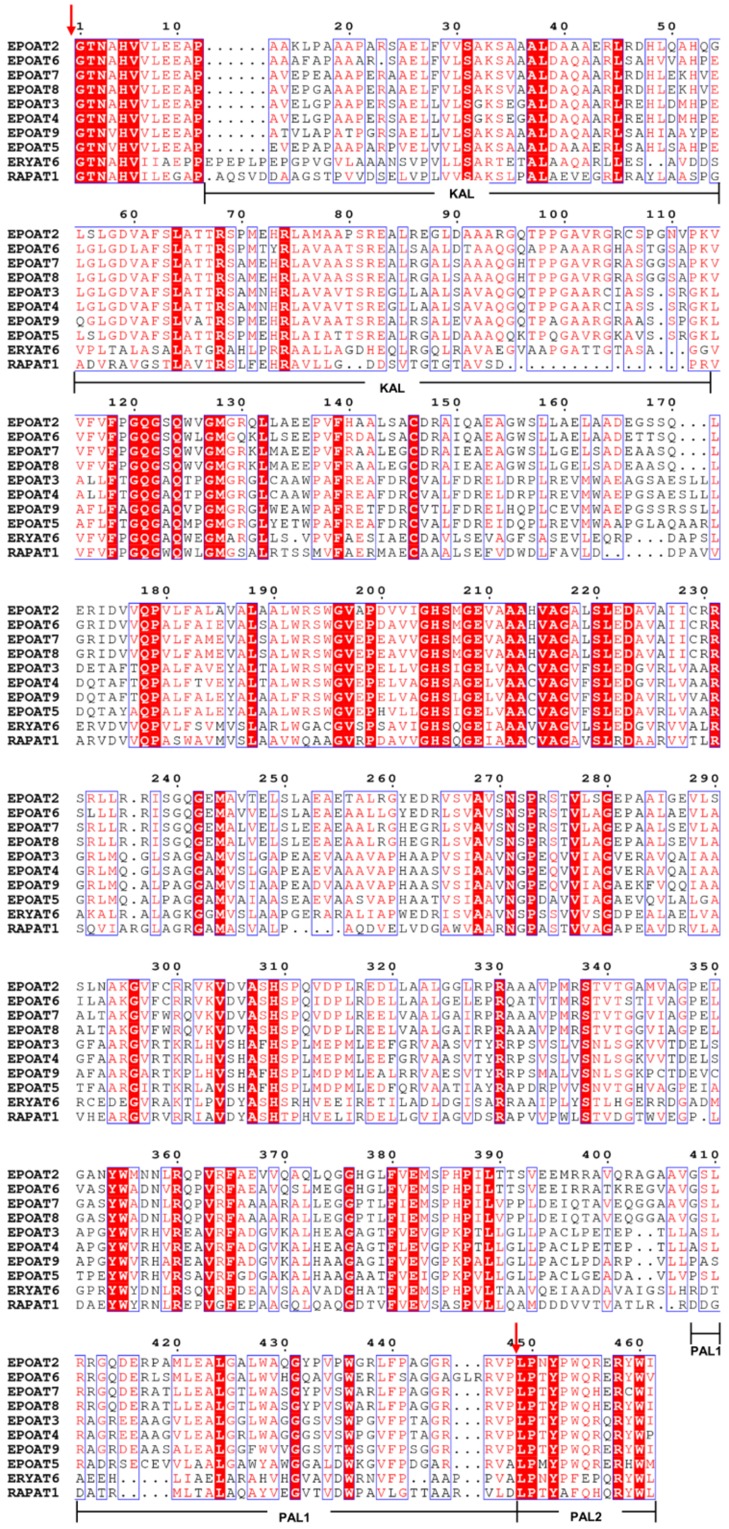

Figure S4. Multiple sequence alignment of AT domains used in this study. Red arrows indicate boundaries for AT swap. EPO, epothilone PKS; ERY, erythromycin PKS; RAP, rapamycin PKS; KAL, KS-AT linker; PAL1, non-conserved N-terminal region in the post-AT linker; PAL2, conserved C-terminal region in the post-AT linker.

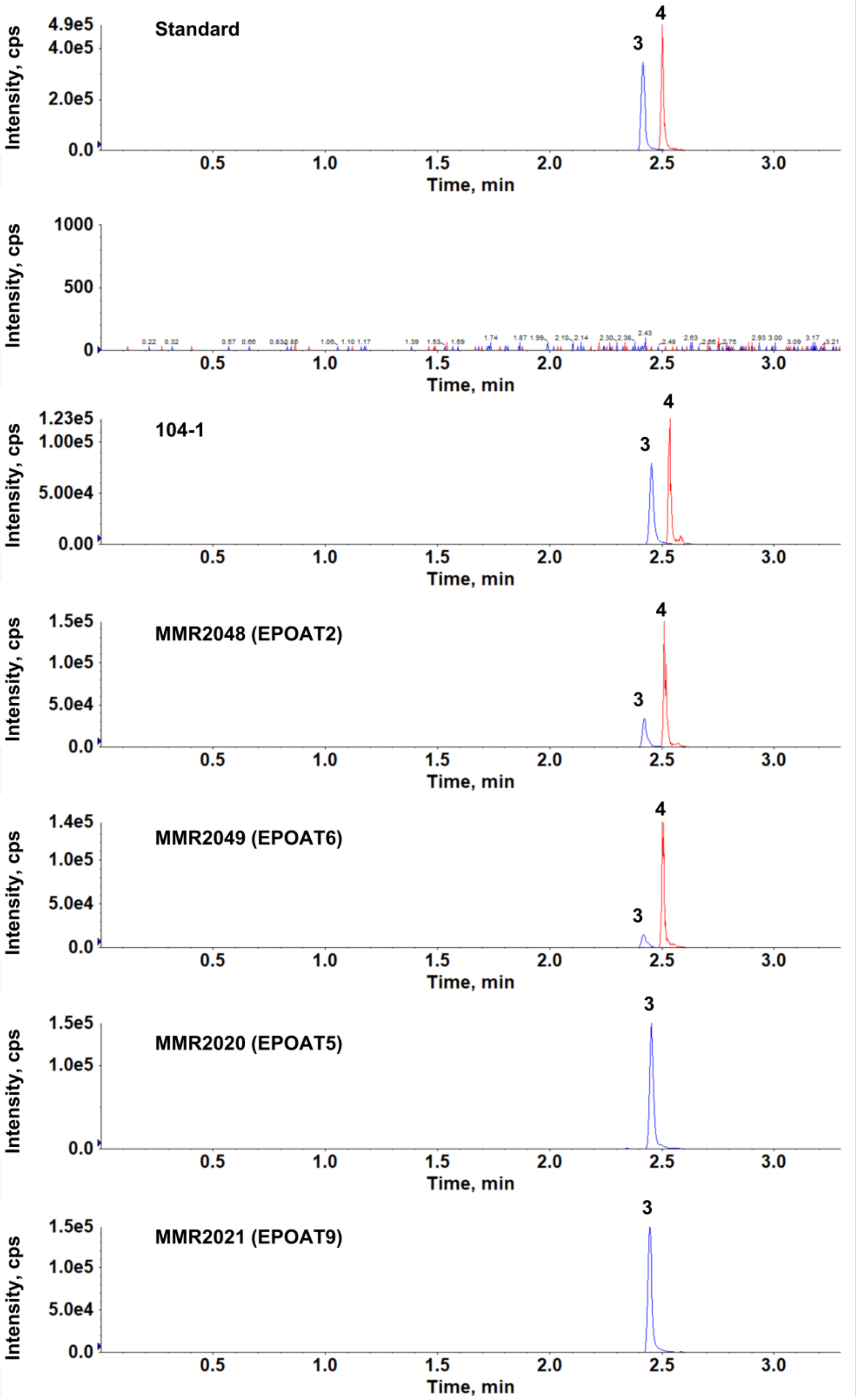

Figure S5. Extracted ion chromatogram (EIC) of LC–MS analyses of epothilone production in some AT-swap mutants to further confirm the production of epothilones. Peaks corresponding to epothilone C (selected for *m/z* = 478.4 and 290.2), epothilone D (selected for *m/z* = 492.4 and 304.2) were shown in blue and red, respectively.

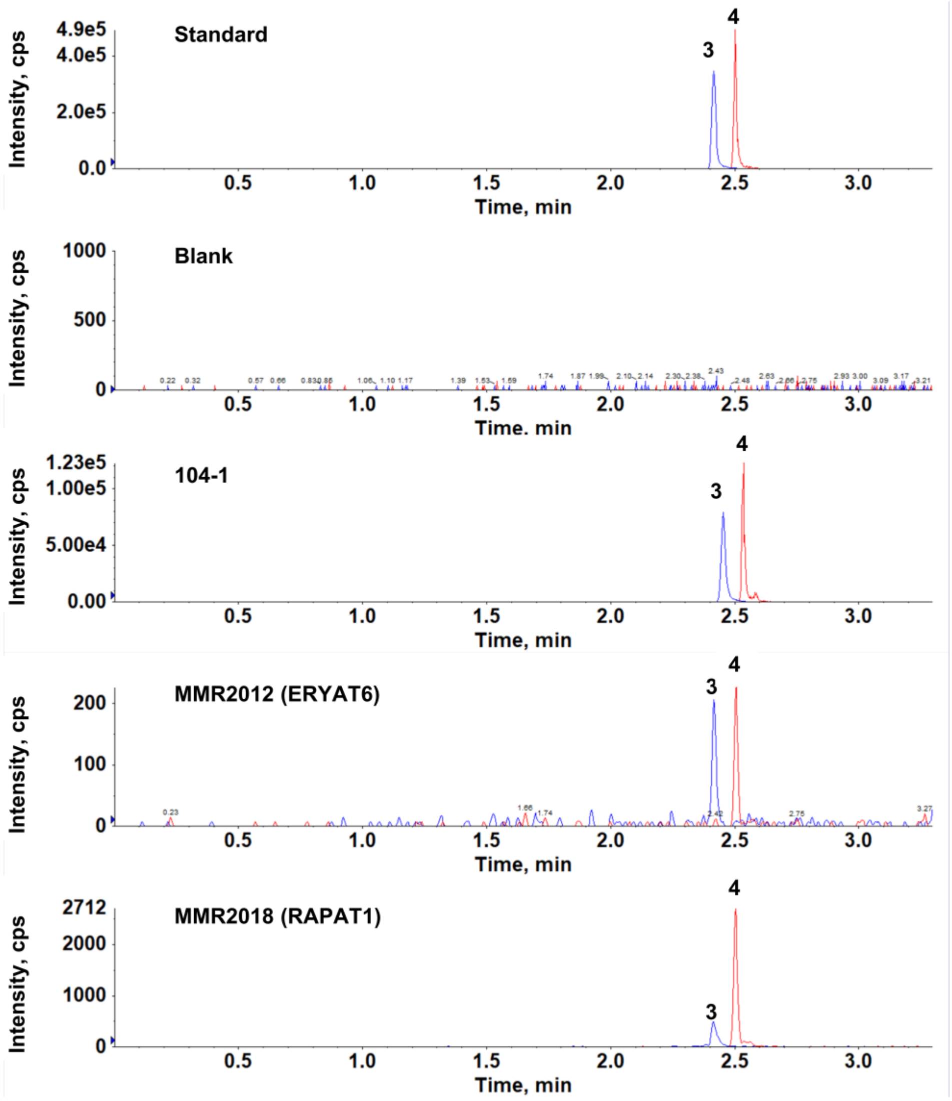

Figure S6. Extracted ion chromatogram (EIC) of LC–MS analyses of epothilone production in AT-swap mutants MMR2012 and MMR2018. Peaks corresponding to epothilone C (selected for *m/z* = 478.4 and 290.2), epothilone D (selected for *m/z* = 492.4 and 304.2) were shown in blue and red, respectively.

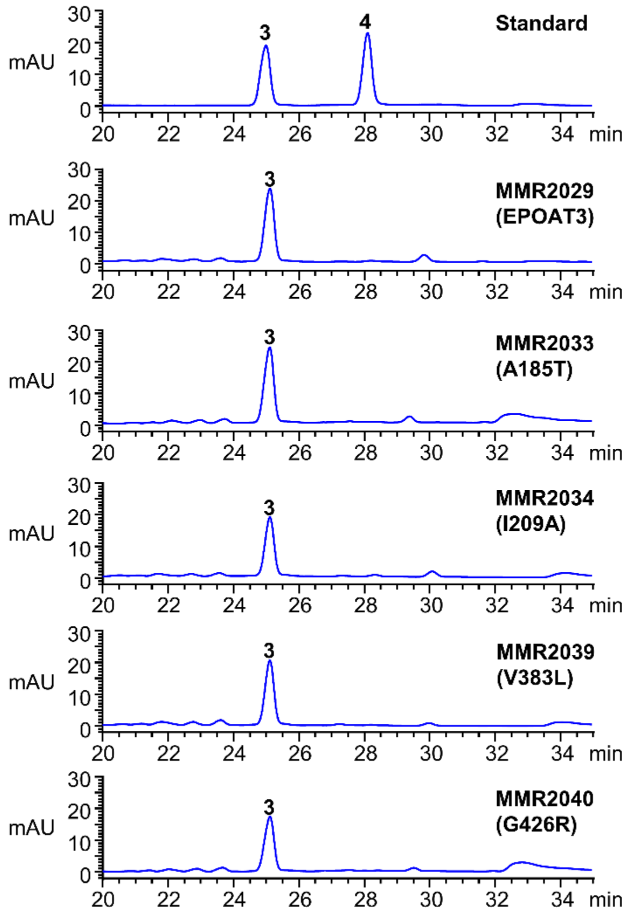

Figure S7. HPLC analysis of the extracts from the parental and site-directed mutant strains.

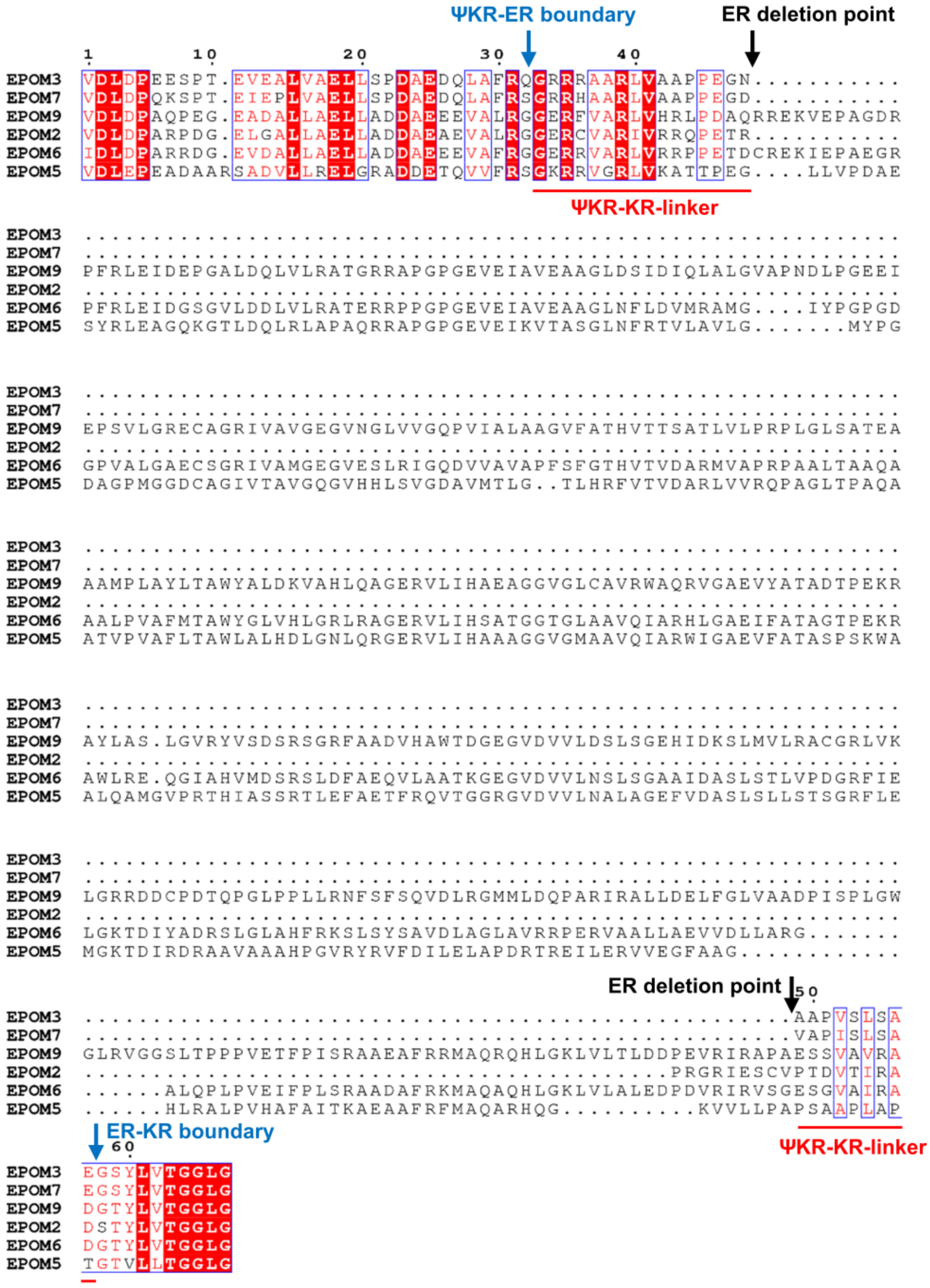

Figure S8. Junctions for ER domain deletion constructs. Black arrows indicate ER domain deletion points. Blue arrows indicate ΨKR-ER and ER-KR boundaries. The ΨKR9-KR9-linker is underlined in red. EPO: epothilone PKS.

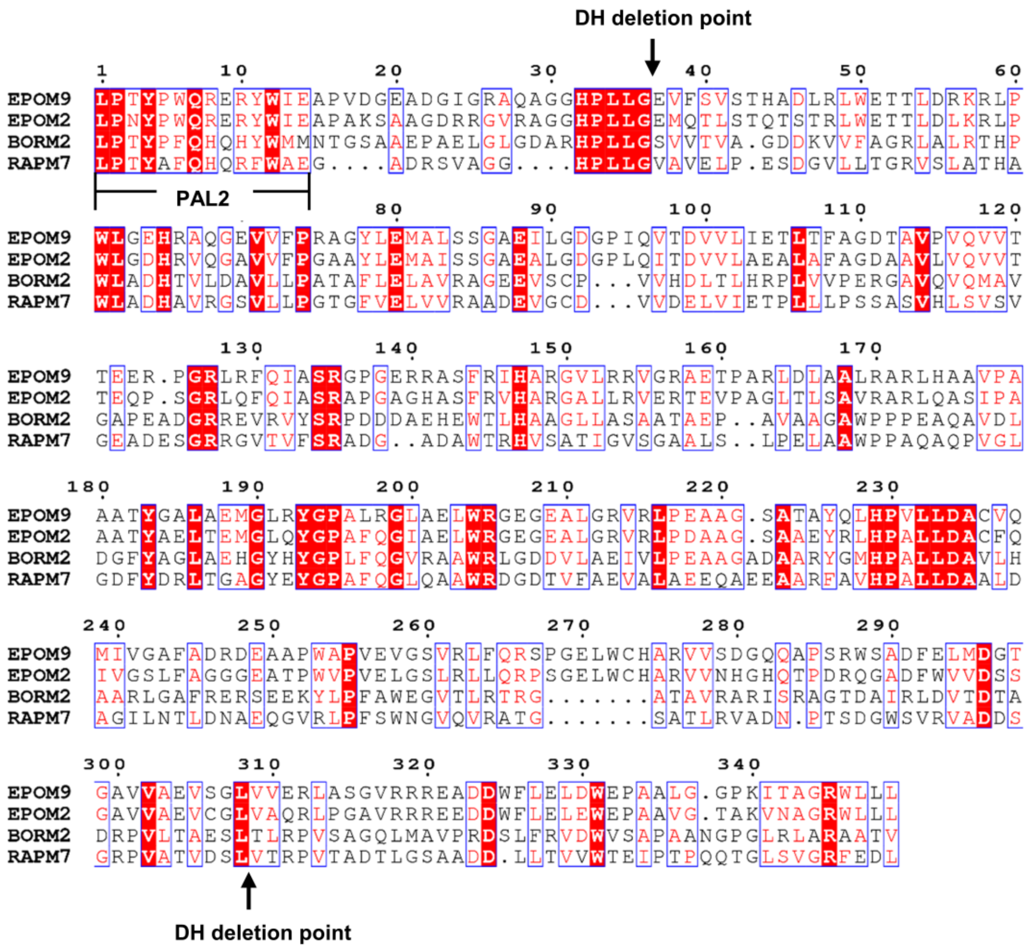

Figure S9. Junctions for DH domain deletion constructs. Arrows indicate deletion points. BOR: borrelidin PKS [1]; RAP: rapamycin PKS.

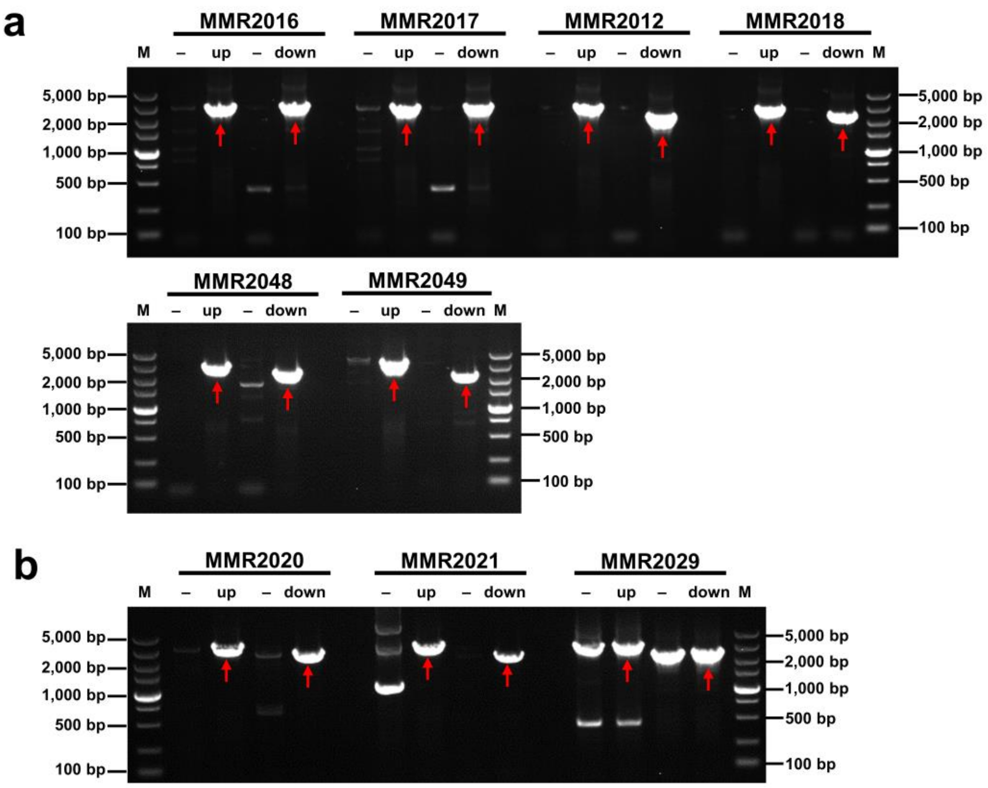

Figure S10. PCR analysis for the confirmation of AT-swap mutants. **a** PCR analysis for the replacement of EPOAT4 with MMCoA-specific ATs, which resulted in MMR2016, MMR2017, MMR2012, MMR2018, MMR2048, and MMR2049. **b** PCR analysis for the replacement of EPOAT4 with MCoA-specific ATs, which resulted in MMR2020, MMR2021, and MMR2029. –, PCR analysis with genomic DNA from *Schlegelella brevitalea* 104-1, which was used as a negative control. Red arrows indicate the expected up-AT4swap and AT4swap-down fragments in seven mutant strains that were amplified by PCR using the primers JM12-F/R and JM20-F/R (Additional file 1: Table S3). The up-AT4swap and AT4swap-down fragments are 2932 bp and 3053 bp long for MMR2016 and MMR2017, 2958 bp and 2284 bp for MMR2012, 2856 bp and 2351 bp for MMR2018, 2917 bp and 2364 bp for MMR2048, 3274 bp and 2199 bp for MMR2049, 3116 bp and 2532 bp for MMR2020, 3108 bp and 2370 bp for MMR2021, and 3125 bp and 2425 bp for MMR2029. The expected DNA fragments were obtained from both 104-1 and MMR2029 (EPOAT3) because EPOAT3 and EPOAT4 differ in only nine amino acids. All PCR products were then sequenced, and the results further confirmed that EPOAT4 in the epothilone biosynthetic gene cluster was successfully replaced with non-native AT domains by double crossover.

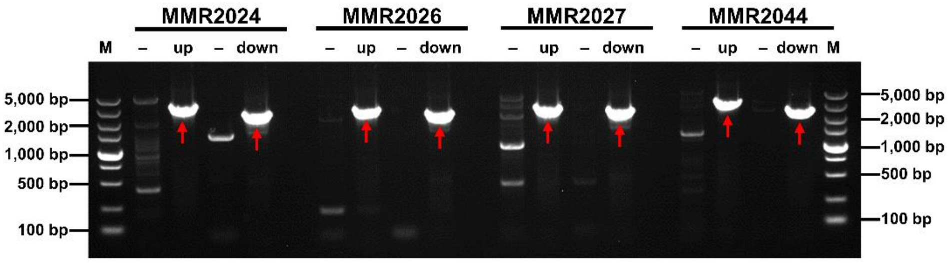

Figure S11. PCR analysis for the confirmation of module-exchange mutants. PCR analysis for the substitution of EPOM4 with modules that harbour MMCoA-specific AT domains, which resulted in MMR2024, MMR2026, MMR2027, and MMR2044. –, PCR analysis with genomic DNA from *Schlegelella brevitalea* 104-1, which was used as a negative control. Red arrows indicate the expected up-M4swap and M4swap-down fragments in four mutant strains by PCR that were generated using the primers JM28-F/R and JM32-F/R (Additional file 1: Table S3). The up-M4swap and M4swap-down fragments are 3274 bp and 2616 bp long for MMR2024, 2908 bp and 2537 bp for MMR2026, 2907 bp and 2670 bp for MMR2027, and 3446 bp and 2502 bp for MMR2044. All PCR products were then sequenced, and the results further confirmed that EPOM4 in the epothilone biosynthetic gene cluster was successfully replaced with non-native modules by double crossover.

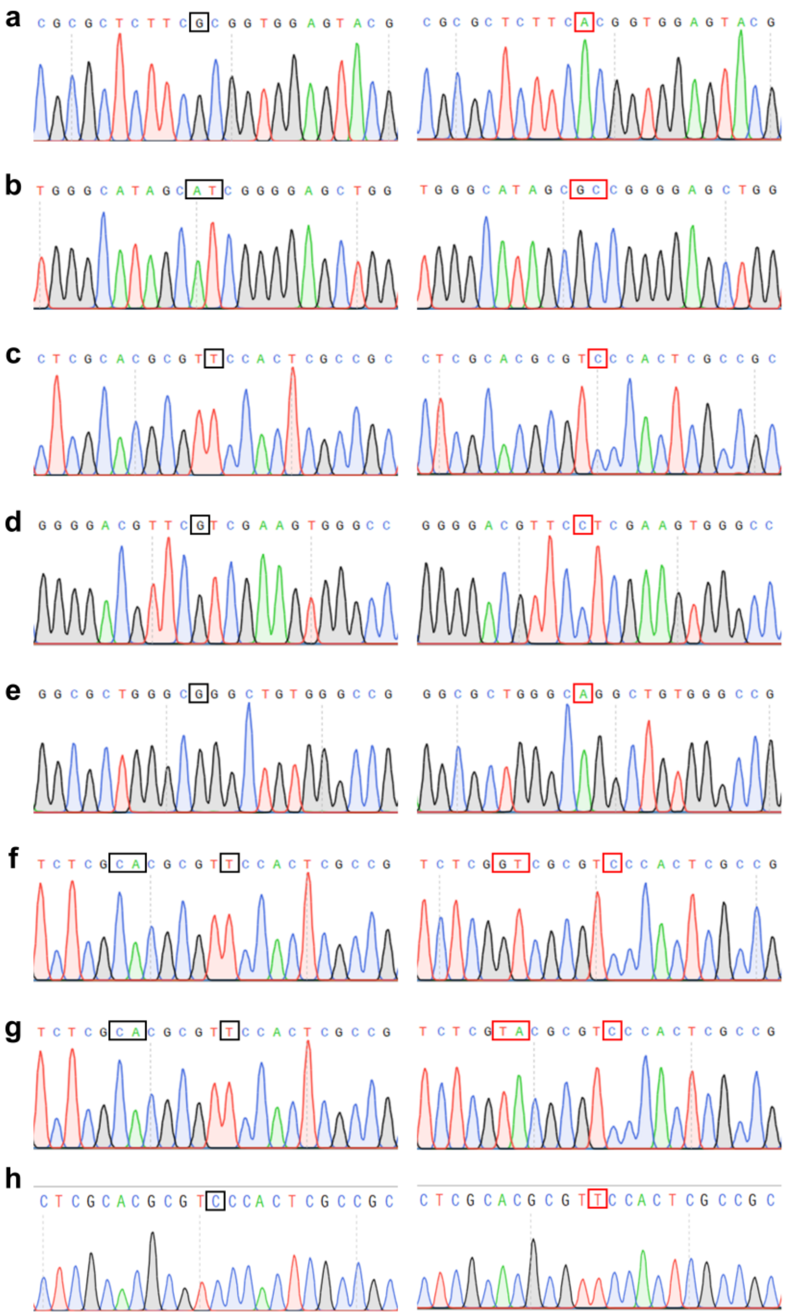

Figure S12. DNA sequences of the PCR products of site-directed mutants. The left column in **a-g** shows DNA sequences of the PCR products of parent strain MMR2029. The left column in **h** shows DNA sequences of the PCR products of parent strain 104-1. The right column shows DNA sequences of the PCR products of site-directed mutants. **a** MMR2029 and MMR2033 (A185T). **b** MMR2029 and MMR2034 (I209A). **c** MMR2029 and MMR2035 (F310S). **d** MMR2029 and MMR2039 (V383L). **e** MMR2029 and MMR2040 (G426R). **f** MMR2029 and MMR2041 (F310S-H308V). **g** MMR2029 and MMR2042 (F310S-H308Y). **h** 104-1 and MMR2055 (S310F). The mutated bases are highlighted with red boxes, and the corresponding bases in MMR2029 and 104-1 are highlighted with black boxes.

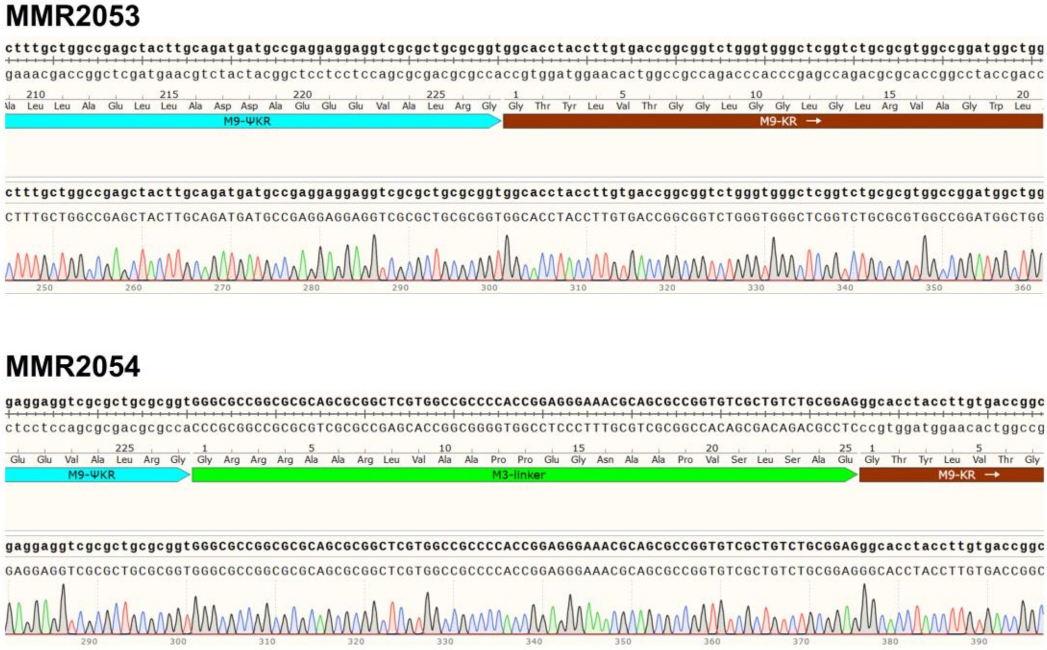

Figure S13. DNA sequences of the PCR products of ΨKR9-KR9-linker deletion mutant (MMR2053) and replacement mutant (MMR2054).

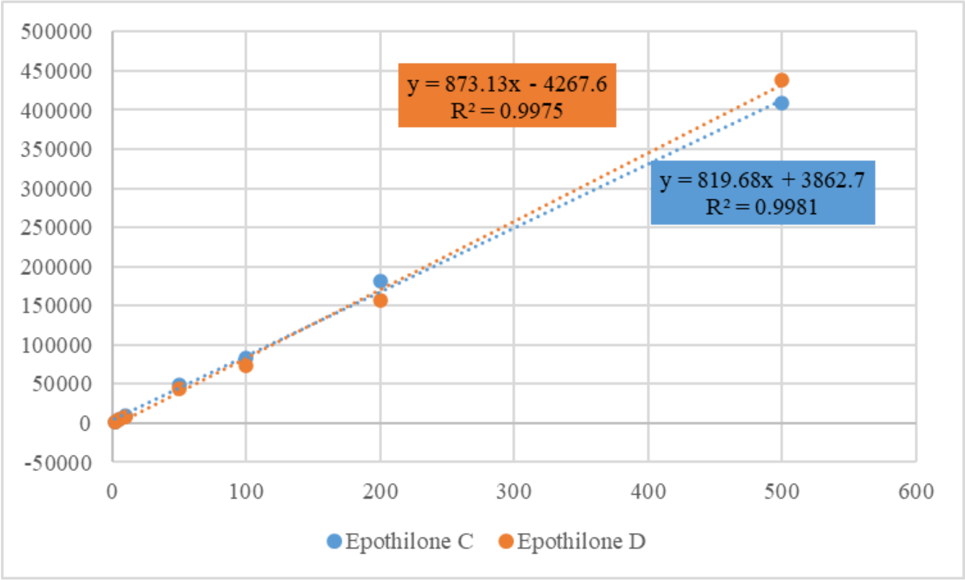

Figure S14. Standard curves calculated based on the peak areas of epothilone standards of different concentrations. Different concentrations of each epothilone component (1 ng/ml, 5 ng/ml, 10 ng/ml, 50 ng/ml, 100 ng/ml, 200 ng/ml, and 500 ng/ml) were used to test the standard curves.

References

1. Hagen A, Poust S, Rond T, Fortman JL, Katz L, Petzold CJ, Keasling JD: **Engineering a Polyketide Synthase for In Vitro Production of Adipic Acid**. *ACS SYNTH BIOL* 2016, **5**(1):21-27.
